# METTL14/miR‐29c‐3p axis drives aerobic glycolysis to promote triple‐negative breast cancer progression though TRIM9‐mediated PKM2 ubiquitination

**DOI:** 10.1111/jcmm.18112

**Published:** 2024-01-23

**Authors:** Hao Wu, Yile Jiao, Xinyi Guo, Zhenru Wu, Qing Lv

**Affiliations:** ^1^ Division of Breast Surgery, Department of General Surgery West China Hospital, Sichuan University Chengdu China; ^2^ Breast Center, West China Hospital Sichuan University Chengdu China; ^3^ Laboratory of Pathology, West China Hospital Sichuan University Chengdu China

**Keywords:** aerobic glycolysis, METTL14/miR‐29c‐3p axis, PKM2, TRIM9, triple negative breast cancer, ubiquitination

## Abstract

The energy metabolic rearrangement of triple‐negative breast cancer (TNBC) from oxidative phosphorylation to aerobic glycolysis is a significant biological feature and can promote the malignant progression. However, there is little knowledge about the functional mechanisms of methyltransferase‐like protein 14 (METTL14) mediated contributes to TNBC malignant progression. Our study found that METTL14 expression was significantly upregulated in TNBC tissues and cell lines. Silencing METTL14 significantly inhibited TNBC cell growth and invasion in vitro, as well as suppressed tumour growth. Mechanically, METTL14 was first found to activate miR‐29c‐3p through m6A and regulate tripartite motif containing 9 (TRIM9) to promote ubiquitination of pyruvate kinase isoform M2 (PKM2) and lead to its transition from tetramer to dimer, resulting in glucose metabolic reprogramming from oxidative phosphorylation to aerobic glycolysis to promote the progress of TNBC. Taken together, these findings reveal important roles of METTL14 in TNBC tumorigenesis and energy metabolism, which might represent a novel potential therapeutic target for TNBC.

## 1 INTRODUCTION

Breast cancer (BC) has become the cancer with the highest incidence rate in the world.^1^, ^2^ Every year, 416,000 new cases of breast cancer occur in China, and 117,000 deaths occur.^3^ Importantly, triple‐negative breast cancer (TNBC) is the most difficult to treat and the highest mortality subtype of breast cancer.^4^, ^5^ Most patients have axillary lymph node metastasis at the time of initial diagnosis.^6^ The current comprehensive treatment including surgery, radiotherapy and systemic drugs can significantly improve the prognosis of patients, but recurrence and distant metastasis are still the main reasons for the failure of treatment of breast cancer.^7^ The situation of prevention and control is urgent.

In recent years, m6A methylation modification as a post‐transcriptional modification has become the most important research direction.^8^ Methylation of m6A has been proven to be widely involved in malignant processes such as tumour cell proliferation, invasion and metastasis.^9^, ^10^ The entire process of initiation and progression of m6A methylation is strictly regulated by related genes, namely, so‐called methyltransferases (writers), effector proteins that recognize methylated target proteins (readers) and demethylases (erasers).^11^ In mammalian cells, methyltransferase‐like 3 (METTL3) and METTL14 are two m6A methyltransferases reported, both of which contain a methyltransferase domain.^11^ These two proteins form a stable heterodimeric core complex of METTL3‐METTL14, which plays a role in the deposition of m6A in mammalian nuclear RNA in cells.^12^ METTL3 is a subunit with catalytic activity, and METTL14 plays a key structural role in substrate recognition.^12^ Research has confirmed that only intervening with METTL14, rather than METTL3, can reduce the level of m6A methylation modification in tumour cells.^13^ In addition, METTL14 knockout resulted in a more severe phenotype than METTL3 knockout, suggesting that METTL14 may have a regulatory function of m6A methylation independent of METTL3.^14^ Under in vitro conditions, METTL14 alone has a higher m6A methylation transfer ability than METTL3 alone, and the catalytic ability is further improved when METTL14 and METTL3 form a heterodimer complex.^15^ Therefore, METTL14 is an important signal for m6A methylation modification. With the deepening of research, it has been found that m6A methylation can occur not only on mRNA, but also on non‐coding RNA such as microRNA (miRNA).^16^, ^17^ Research has confirmed that m6A methylation can cause methylation of pri‐miRNA, and the pri‐miRNA that undergoes m6A methylation can be recognized and processed by Di George syndrome critical region gene 8 (DGCR8), promoting the maturation of miRNA.^18^ Decreasing m6A methylation of pri‐miRNA will decrease the binding effect with DGCR8, resulting in a decrease in miRNA expression in mature bodies.^19^ While METTL14 interacts with DGCR8 and is RNA mediated, silencing METTL14 can cause a significant increase in pri‐miRNA expression levels, affecting miRNA expression levels in mature bodies.^20^ However, m6A modification of miRNA mediated by METTL14 regulates the malignant progression of breast cancer remains unclear.

Warburg effect is the main way of adenosine triphosphate (ATP) production in tumour cells, and its essence is that the mitochondrial oxidative phosphorylation of glucose changes into aerobic glycolysis.^21^, ^22^, ^23^ Pyruvate kinase (PK) is the ultimate rate‐limiting enzyme of glycolysis.^24^ PKM2 is a key enzyme in Warburg effect.^25^, ^26^ PKM2 has two conformations in cells: high enzyme activity tetramer and low enzyme activity dimer.^27^ PKM2 is highly expressed in tumour cells in the form of low enzyme activity dimer, which helps to combine carbon atoms into bioactive substances more quickly, promote the rapid generation of energy from glycolysis intermediates and flow into collateral pathways and synthesize nucleic acids, amino acids and lipids without accumulating reactive oxygen species to provide energy and metabolic substances needed for the malignant growth of breast cancer cells.^28^, ^29^ Therefore, the low enzymatic activity dimer conformation of PKM2 is a necessary condition to ensure the Warburg effect. Zhou et al. found that in breast cancer, the dimer conformation of PKM2 significantly promotes Warburg effect and cell proliferation of breast cancer cells.^30^ Hypoxia can induce the expression of hypoxia inducible factor‐1α (HIF1α) through transcription to inhibit the expression of miR‐16‐5p, and promote the expression of YTH domain family 1 (YTHDF1), and thus enhance the expression of PKM2 through m6A methylation to enhance tumour glycolysis and ultimately increase the tumorigenesis and metastasis ability of breast cancer cells.^31^ However, whether PKM2 is involved in glycolytic dysregulation of TNBC remains unclear.

In this study, we explored the molecular mechanism of METTL14‐mediated m6A to regulate miRNA, and analysed the role and the specific mechanism of PKM2 in METTL14‐mediated m6A affecting TNBC aerobic glycolysis and malignant progression.

## 2 MATERIALS AND METHODS

### 2.1 Patient samples

A total of 21 samples from patients with TNBC (tumour tissues and paired normal adjacent tissues) and 61 breast cancer samples (tumour tissues and paired normal adjacent tissues) were retrospectively obtained from the Department of Breast Surgery, West China Hospital, Sichuan University between May 2018 and July 2021. The primary cancer tissues and the paired normal adjacent tissues were immediately snap‐frozen and stored at liquid nitrogen until use for mRNA and protein analyses. The present study was approved by the Institutional Review Board of West China Hospital, Sichuan University. All patients provided written informed consent to participate in the study.

### 2.2 Cell lines

The Human TNBC cell lines (Hs578T, MDA‐MB‐231, HCC1806, BT20 and MDA‐MB‐157), MCF‐10A (human normal mammary epithelial cell line) were purchased from the American Type Culture Collection. TNBC cells were cultured in DMEM (Gibco) containing 10% fetal bovine serum (Gibco). MCF‐10A cells were cultured in MEMG medium (Gibco) supplemented with 10% fetal bovine serum (Gibco). Cells were cultured in a humidified atmosphere containing 5% CO_2_ at 37°C.

### 2.3 Cell transfection

Short hairpin (sh)RNA targeting TRIM9 for knockdown (Sh‐TRIM9), shRNA targeting METTL14 for knockdown (METTL14‐KD) and the respective negative controls (NC) (Shanghai GenePharma). The shRNA sequence Sh‐TRIM9 and METTL14‐KD were constructed into the pLVX‐shRNA2‐BSD lentiviral vector (Takara Bio USA, Inc.). Overexpression TRIM9 (Oe‐TRIM9) and overexpression METTL14 (Oe‐TRIM14), lentiviruses (System Biosciences, LLC) were constructed into the lentiviral vector pCDH‐EF1α‐MCS‐T2A‐Puro (System Biosciences, LLC). Cells in each well were transfected with 100 nM miR‐29c‐3p mimic or 200 nM miR‐29c‐3p inhibitor.

### 2.4 Bioinformatics database analysis

The UALCAN database (http://ualcan. path.uab.edu/cgi‐bin/ualcan‐res.pl) was used to analyse the expression of METTL14 and TRIM9 in the TCGA and CPTAC datasets. The Human Protein Atlas (https://www.proteinatlas.org/) was used to analyse the expression of METTL14 in immunohistochemistry results.

### 2.5 Immunohistochemistry

BC and TNBC tissue sections were rehydrated in xylene and alcohol, and then rehydrated at 37°C with 3% H_2_O_2_ for 30 min. Next, all slices were incubated with goat serum for 15 min, and then washed with TBST three times. Ten minutes each time. Then, the sections were incubated overnight with METTL14 (1: 1000, Abcam) and Ki‐67 (1: 1000, Abcam) at 4°C. Next, the sections were incubated with anti‐rabbit secondary IgG antibody at 37°C for 30 min. DAB (Boster) was used for visual colour rendering of the signal.

### 2.6 Western blot analysis

Total protein was isolated from TNBC samples (tumour tissues, paired normal adjacent tissues and cell lines) using RIPA lysis buffer (Beyotime Institute of Biotechnology). Total protein was separated by 10% SDS‐PAGE (25 μg protein per lane), and the protein was transferred with PVDF membrane (MilliporeSigma). Blocking of PVDF membrane was performed with 5% nonfat milk powder (Beyotime). To detect the expression of PKM2 protein in different conformations, the total protein extracted from the sample was not thermal denatured and was denatured using a non‐denatured gel sample loading buffer (Beyotime). Next, protein electrophoresis was performed using native PAGE running buffer (Beyotime). The PVDF membranes were probed at 4°C overnight with antibodies against METTL14 (1:1000; Abcam), PKM2 (ProteinTech Group, Inc.), DGCR8 (1:1000; Abcam), TRIM9 (1:1000; Abcam) and β‐actin (1:5000; Abcam). Finally, protein expression was analysed by chemiluminescence reagents (Hyperfilm ECL).

### 2.7 RNA extraction and reverse transcription‐quantitative (RT‐qPCR)

Total RNA was extracted from TNBC samples (tumour tissues, paired normal adjacent tissues and cell lines) using TRIzol reagent (Takara Bio). The extracted RNA was reverse transcribed to cDNA with PrimeScript RT Reagent (Takara Bio, Inc.). Real‐time qPCR was performed with a Prime‐Script® RT Reagent Kit (Takara Bio, Inc.) and a LightCycler system (Roche Diagnostics) was used for detection. GADPH and U6 are used as internal references. The sequences used in this study were listed in Table S1.

### 2.8 Co‐immunoprecipitation (Co‐IP)

Cells were lysed using a RIPA lysis buffer containing a protease inhibitor (Beyotime). Next, 3 μg antibody was added to the cell lysate, mixed well and incubated at 4°C overnight. Then, 20 μL protein A/G PLUS‐Agarose beads (Santa Cruz Biotechnology) were added to the mixture and cultured on a spinner at 4°C for 4 h. The bead‐antibody‐protein complex was gently washed three times with pre‐cooled PBS and then boiled for western blotting. PKM2 (1:1000; ProteinTech Group, Inc.) and TRIM9 (1:1000; Abcam) and DGCR8 (1:1000; Abcam).

### 2.9 RNA immunoprecipitation

For the m6A RNA‐binding assays, RNA was chemically fragmented to −100 nt by Magna MeRIP m6A Kit (Millipore) Then, the fragmented RNA was incubated with magnetic beads conjugated with m6A antibodies (Millipore) for immunoprecipitation. The enrichment of mRNA containing m6A was analysed by RT‐qPCR and normalized to input.

### 2.10 Ubiquitination assay

TNBC cells were lysed by centrifuge tubes in 1% SDS RIPA buffer in a mixed bath of ice water. Cell lysates were treated at room temperature with protein A/G Plus‐ agarose (Santa Cruz Biotechnology) for 1 h. Next, cell lysates was incubated with IgG (1:500; ProteinTech Group, Inc.) overnight at 4°C. Nuclear pellet are collected by centrifugation at 10,000*g* at 4°C for 5 min, followed by washing with protein A/g Plus‐AGAR beads four times. Proteins were purified through gradient SDS‐PAGE separation. According to the regimen of western blotting, anti‐PKM2 (1:1000; Abcam) or anti‐ubiquitin antibody (1:500; Abcam) were used for immunoblotting.

### 2.11 Glucose uptake assay

TNBC cells were incubated in DMEM without any L‐glucose or phenol red for 8 h. The amount of glucose in the media was measured using a Glucose Colorimetric Assay kit (BioVision, Inc.). Fresh DMEM without any L‐glucose and phenol red was used for the negative control.

### 2.12 Intracellular pyruvate assay, lactate assay and ATP assay

TNBC cells were incubated in phenol red‐free DMEM without FBS for 4 h. Next, a pyruvate assay kit (Solarbio), lactate assay kit (Solarbio) and ATP assay kit (Solarbio) were used to measure intracellular concentrations of pyruvate, lactate and ATP, respectively.

### 2.13 Oxygen consumption rate (OCR) and extracellular acidification rate (ECAR) assay

TNBC cells were plated in XF96 cell culture plates (Seahorse Bioscience) and cultured for 8 h. Next, the cells were balanced without CO_2_ for 1 h by buffered DMEM without bicarbonate prior to XF measurement. Determination of glycolysis rate in XF basal medium without phenol red was calculated. Inhibitors were added: 0.5 μM rotenone/antimycin A (each) and 50 mM 2‐deoxyglycine. Mitochondrial stress tests were performed in XF basal medium containing 10 mM glucose, 1 mM sodium pyruvate and 2 mM L‐glutamine. Inhibitors were added: 1 μM oligomycin, carbonyl cyanide 4‐ (trifluoromethoxy) 1 μM phenylhydrazine and 0.5 Μm rotenone/antimycin A (each).

### 2.14 Data filtering and mapping

Use the following websites and software to process and visualize data: FastQC (http://www.bioinformatics.babraham.ac.uk/projects/fastqc/), miRBase21.0 (http://www.mirbase.org/) And piRNAcluster (http://www.smallrnagroup.uni‐mainz.de/piRNAclusterDB.html). National Center for Biotechnology Information database (https://www.ncbi.nlm.nih.gov/). Genomic tRNA Database (GtRNAdb, http://gtrnadb.ucsc.edu/) and tRFdb (http://genome.bioch.virginia.edu/trfdb/). Rfam (http://rfam.xfam.org/).

### 2.15 Differentially expressed miRNA identification

|Log_2_ (fold change)| >1 and false discovery rate (FDR) <0.05 were considered to be significantly different (differentially expressed (DE) miRNAs).

### 2.16 Functional enrichment analysis of target genes

The following websites and software were used for bioinformatics analysis of the data: Discovery 6.8 Bioinformatics Tool (DAVID 6.8), Gene Ontology (GO, http://www.geneontology.org/) and Kyoto Encyclopedia of Genes and Genomes (KEGG, http://www.kegg.jp/). The critical threshold is set to *p* value <0.05.

### 2.17 Immunofluorescence of cells

TNBC cells were seeded and cultured on coverslips. Then, slide containing cells were fixed at room temperature with 4% paraformaldehyde for 30 min, and permeated with 0.25% Triton X‐100 solution for 20 min. Cell slides were washed with PBS three times for 5 min each time, then blocked with 5% bovine serum albumin at room temperature for 1 h. Coverslips were incubated with TRIM9 (1:200, Abcam, USA) antibodies overnight at 4°C. After washing with PBS three times for 5 min each time, coverslips were incubated with a secondary antibody and DAPI.

### 2.18 miRNA prediction

TargetScan (http://www.targetscan.org), Oncomir (http://www.oncomir.org/) and miRWalk (http://mirwalk.umm.uni‐heidelberg.de/) were used to predict miRNA targets and conserved sites bound by miR‐29c‐3p.

### 2.19 Dual luciferase reporter assay

Wild‐type (WT) TRIM9‐3'UTR and mutant (MUT) TRIM9‐3'UTR oligonucleotides containing the putative binding site of miR‐29c‐3p were cloned into the firefly luciferase‐expressing pMIR‐REPORT vector (Obio Technology Corp). These constructs were cotransfected with miR‐29c‐3p mimics, miR‐29c‐3p inhibitor and miRNA‐NC. After 48 h of transfection, luciferase activity was determined using the Dual‐Luciferase Reporter Assay kit (Promega Corporation).

### 2.20 Cell Counting Kit‐8 (CCK‐8) assay

TNBC cells were transfected and then inoculated into 96‐well plates (2 × 10^3^ cells/well). After incubation, CCK‐8 assay reagent (Dojindo Molecular Technologies, Inc.) was added to each well containing cells and plates were incubated. The absorbance of each pore at 450 nm was recorded to analyse cell proliferation.

### 2.21 5‐Ethynyl‐2′‐deoxyuridine (EdU) assay

TNBC cells were transfected and inoculated into 96‐well plates (1 × 10^3^/well). Then, the slide containing cells were fixed at room temperature with 4% paraformaldehyde for 30 min, and permeated with 0.25% Triton X‐100 solution for 20 min. EdU (50 μM), 1× ApolloR reaction cocktail (100 μL) and 1× DAPI (100 μL) to 96‐well plates respectively.

### 2.22 Cell migration and invasion assays

TNBC cells (1 × 10^5^ cells/well) were inoculated into the upper compartment of the Transwell chamber containing DMEM with 1% FBS. Next, 500 μL of DMEM containing 10% FBS was added to lower chamber. After incubation for 48 h, cells were fixed with methanol for 30 min and stained with 0.1% crystal violet for 20 min. For the invasion assay, the inserts were precoated with 1 mg/mL Matrigel (Corning, Inc.).

### 2.23 Cell apoptosis

TNBC cells (1 × 10^5^ cells) were collected and incubated with 5 μL Annexin V‐FITC (Biogot Technology) and 5 μL propidium iodide solution (Biogot Technology) at room temperature for 15 min. Then, cells were suspended in 400 μL binding buffer (Biogot Technology Co.). Cell apoptosis progression was analysed using flow cytometry (FACSAria; BD Biosciences).

### 2.24 Mouse xenograft tumour model

All animal experiments were performed in accordance with the institutional guidelines and were approved by the Committee on the Ethics of Animal Experiments of West China Hospital of Sichuan University. Female BALB/c‐nu mice were purchased from Chengdu Dossy Experimental Animals Co., LTD (Chengdu, China) and fed at the Experimental Animal Center of West China Hospital of Sichuan University. After transfection for 36 h, TNBC cells (5 × 10^6^) were subcutaneously injected into the mammary fat pads of the female athymic nude mice. Tumour volume was calculated according to the following formula: Volume = (width^2^ × length)/2.

### 2.25 Statistical analysis

All data were analysed using SPSS 22.0 software (SPSS Inc., Chicago, IL, USA) or GraphPad Prism version 7.0 (CA, USA). Data were presented as the means ± standard deviation (SD). For two‐group comparisons, the unpaired two‐tailed Student's *t*‐test was performed; for comparisons between more than two groups, one‐way or two‐way ANOVA followed by Tukey's post hoc test was performed. Correlation between miR‐29c‐3p and TRIM9 expression was evaluated using Spearman's correlation analysis. The threshold for statistical significance was *p* < 0.05.

## 3 RESULTS

### 3.1 METTL14 is upregulated in BC and TNBC

**FIGURE 1:**
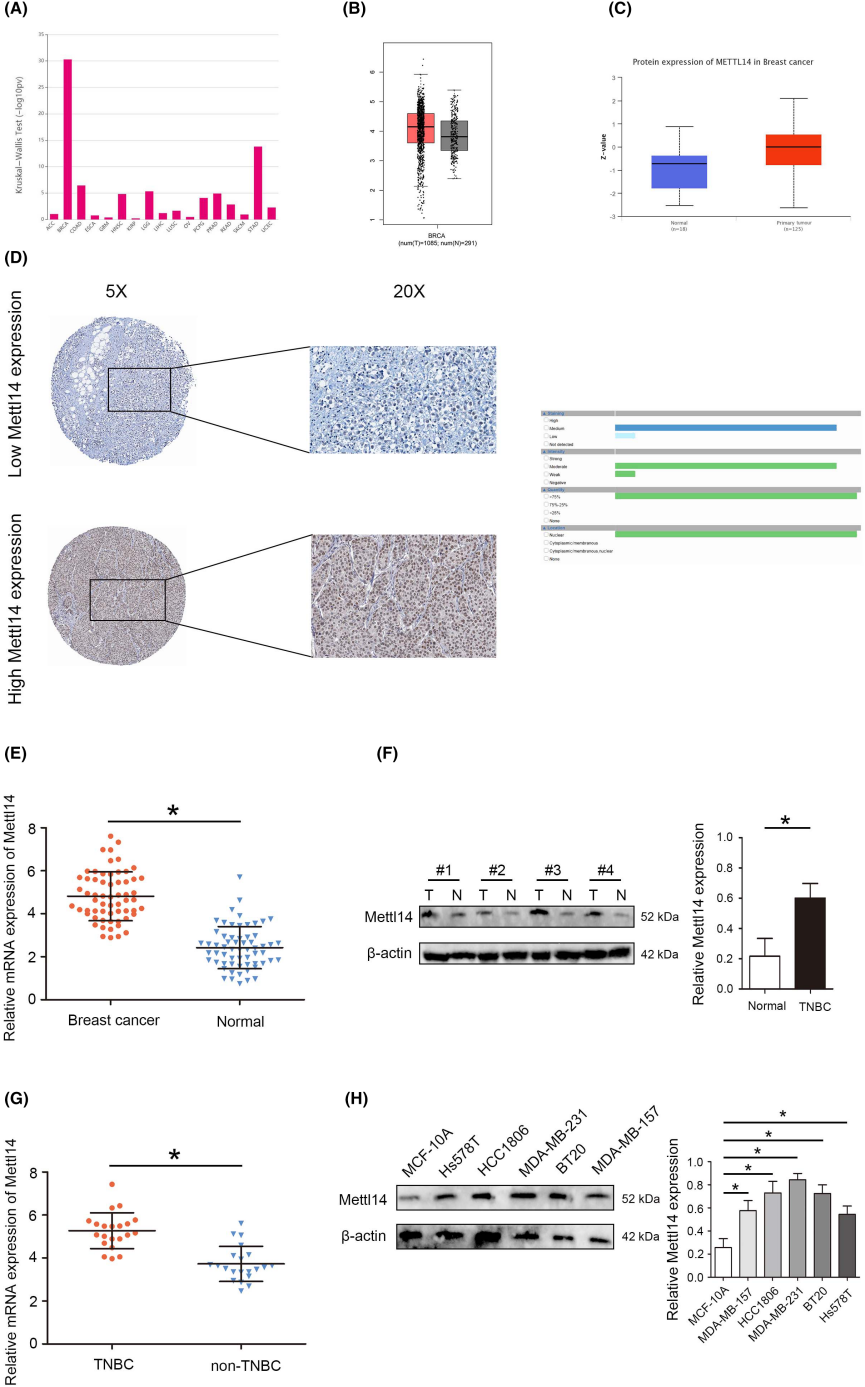
Expression profile of methyltransferase‐like protein 14 (METTL14) in breast cancer (BC) and triple‐negative breast cancer (TNBC). (A) mRNA expression of METTL14 in TCGA database. (B) mRNA expression of METTL14 in the GEPIA database. (C) Protein expression of METTL14 in BC. (D) The expression of METTL14 in BC detected by immunohistochemistry. (E) mRNA expression of METTL14 in BC detected by RT‐qPCR. (F) Protein expression of METTL14 in TNBC detected by western blot. (G) mRNA expression of METTL14 in TNBC detected by RT‐qPCR. (H) Protein expression of METTL14 in TNBC cells detected by western blot *p < 0.05.

First, we analysed the mRNA level of METTL14 in UALCAN database, and found that METTL14 mRNA was highly expressed in BC tumour tissues (Figure 1A–C). Immunohistochemical results also confirmed that METTL14 was highly expressed in BC tissues (Figure 1D). In tissue samples collected from patients with BC, RT‐qPCR analyses suggested that the expression of METTL14 in BC tissues was higher than that in adjacent tissues (Figure 1E). Moreover, METTL14 expression was significantly high expression in TNBC tissues compared with adjacent tissues, and RT‐qPCR results also confirm the above conclusion (Figure 1F,G). Western blot analyses suggested that the expression of METTL14 in TNBC cell lines was significantly higher than that in the normal breast cell line (Figure 1H).

### 3.2 Decreased METTL14 inhibits TNBC cell proliferation and induces TNBC cell apoptosis

**FIGURE 2:**
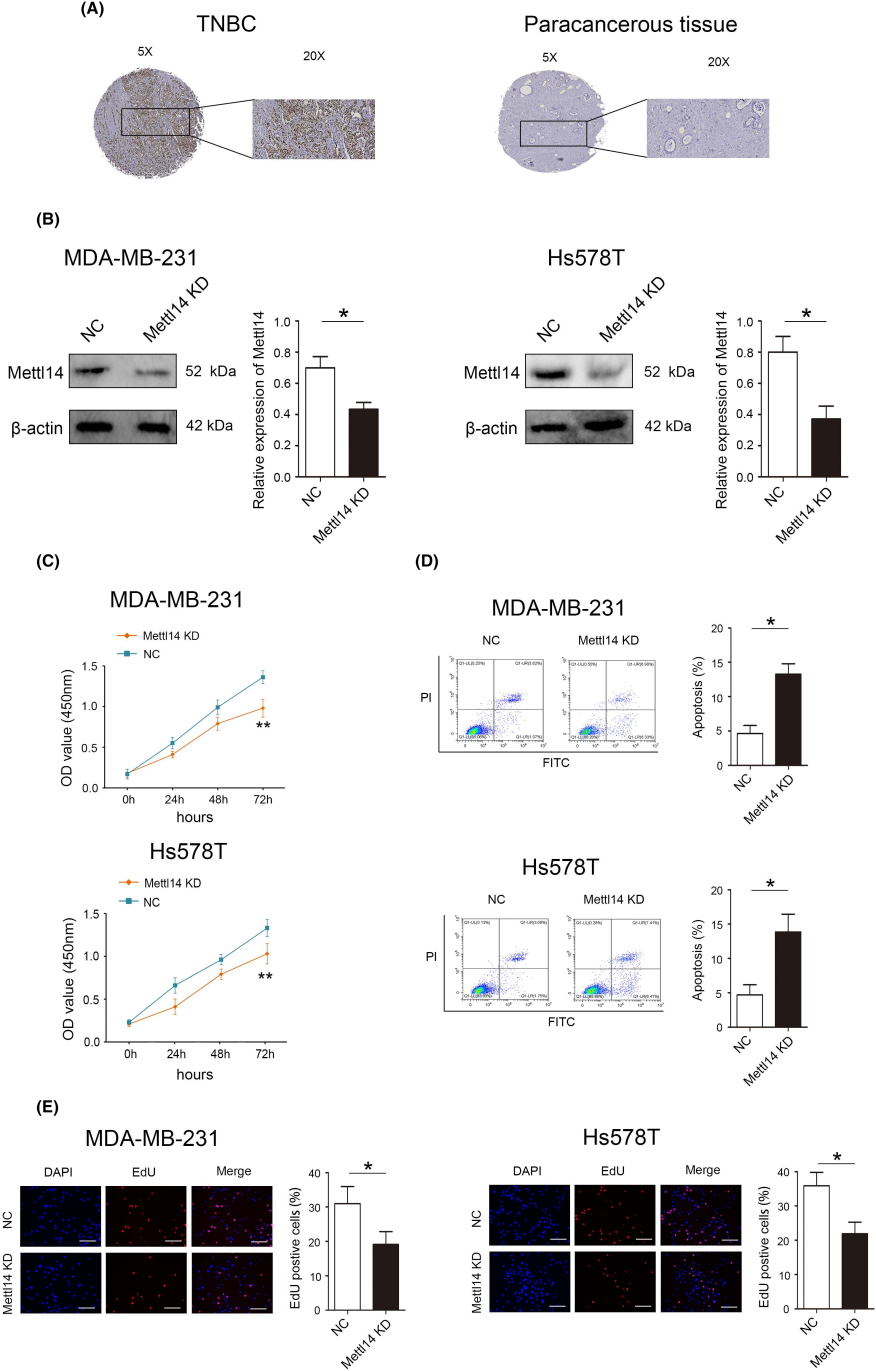
Methyltransferase‐like protein 14 (METTL14) significantly suppresses cell proliferation and promotes apoptosis in triple‐negative breast cancer (TNBC) cell lines. (A) The expression of METTL14 in TNBC tissues and adjacent tissues detected by immunohistochemistry. (B) Protein expression of METTL14 in MD‐MBA‐231 and Hs578T cells detected by western blot. (C) Effect of METTL14 KD on MD‐MBA‐231 and Hs578T cell proliferation detected by Cell Counting Kit‐8 (CCK‐8) assay. (D) Effect of METTL14 KD on MD‐MBA‐231 and Hs578T cell apoptosis detected by Flow cytometry. (E) Effect of METTL14 KD on MD‐MBA‐231 and Hs578T cell proliferation detected by EdU uptake assay. *p < 0.05, **p < 0.01. Scale bars, 50 μm.

In the TNBC samples we collected, immunohistochemical results also confirmed that METTL14 was highly expressed in TNBC tissues (Figure 2A).To further verify the role of METTL14 in TNBC biological behaviour, gain‐of‐function experiments were performed in MDA‐MB‐231 and Hs578T cell lines. First, we effectively reduced the expression of METTL14 in MDA‐MB‐231 and Hs578T (Figure 2B). Decreased METTL14 expression significantly inhibited cell proliferation, as determined by CCK8 assays in MDA‐MB‐231 and Hs578T (Figure 2C). Flow cytometry assay results indicate that decreased METTL14 expression promoted the apoptosis rate of MDA‐MB‐231 (4.69% vs. 13.31%) and Hs578T (4.84% vs. 13.82%) (Figure 2D). As shown in Figure 2E, the number of MDA‐MB‐231 and Hs578T cells incorporating EdU in the METTL14 low expression group was less than the control group.

### 3.3 Decreased METTL14 represses TNBC cells migration, invasion and growth in vitro and in vivo

**FIGURE 3:**
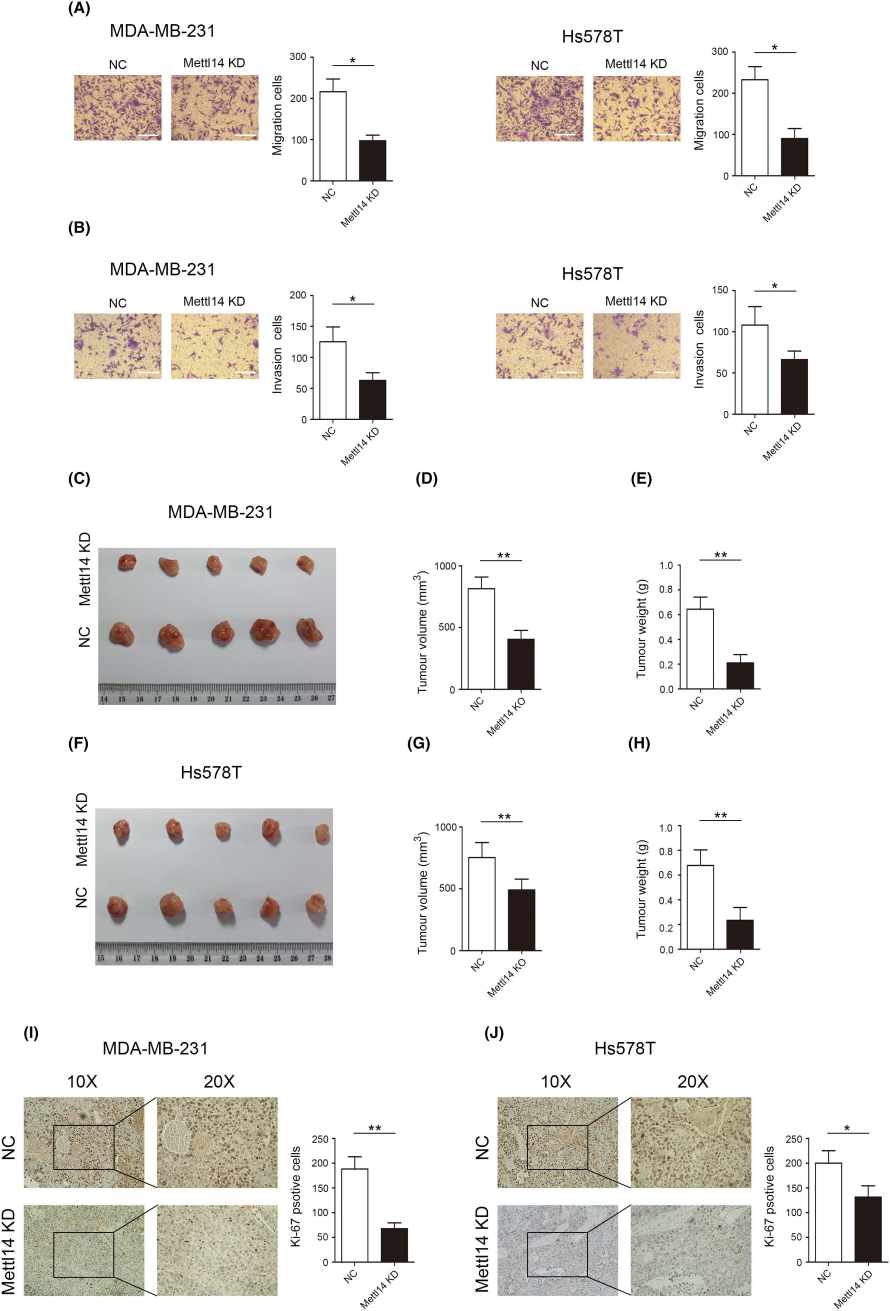
Methyltransferase‐like protein 14 (METTL14) significantly suppresses cell migration, invasion and cell proliferation in vitro and in vivo. (A) Effect of METTL14 KD on MD‐MBA‐231 and Hs578T cell migration detected by transwell assays in vitro. (B) Effect of METTL14 KD on MD‐MBA‐231 and Hs578T cell invasion detected by transwell assays in vitro. (C) Representative images of excised xenograft tumours grown on nude mice from MDA‐MB‐231 cells in vivo. (D) Xenograft tumour volume in each group derived from MDA‐MB‐231 cells in vivo. (E) Xenograft tumour weight in each group derived from MDA‐MB‐231 cells in vivo. (F) Representative images of excised xenograft tumours grown on nude mice from Hs578T cells in vivo. (G) Xenograft tumour volume in each group derived from Hs578T cells in vivo. (H) Xenograft tumour weight in each group derived from Hs578T cells in vivo. (I) Effect of METTL14 KD on Ki‐67 expression in MDA‐MB‐231 cells detected by immunohistochemistry in vivo. (J) Effect of METTL14 KD on Ki‐67 expression in Hs578T cells detected by immunohistochemistry in vivo. *p < 0.05, **p < 0.01. Scale bars, 50 μm.

Furthermore, transwell assay suggested that decreasing the expression of METTL14 significantly inhibited the migration of MDA‐MB‐231 and Hs578T cells (Figure 3A). In addition, transwell assay indicated that decreasing the expression of METTL14 significantly inhibited the invasion of MDA‐MB‐231 and Hs578T cells (Figure 3B). More importantly, the tumour volume and weight in the METTL14 low expressed group were much smaller compared with the control group in MDA‐MB‐231 cells (Figure 3C–E). Similar effects of METTL14 were observed in the Hs578T cells (Figure 3F–H). The results of immunohistochemistry indicated that reducing METTL14 expression inhibits the expression of Ki‐67 in MDA‐MB‐231 and Hs578T cells (Figure 3I,J).

### 3.4 METTL14‐dependent m6A methylation modulates the processing of miR‐29c‐3p by DGCR8

**FIGURE 4:**
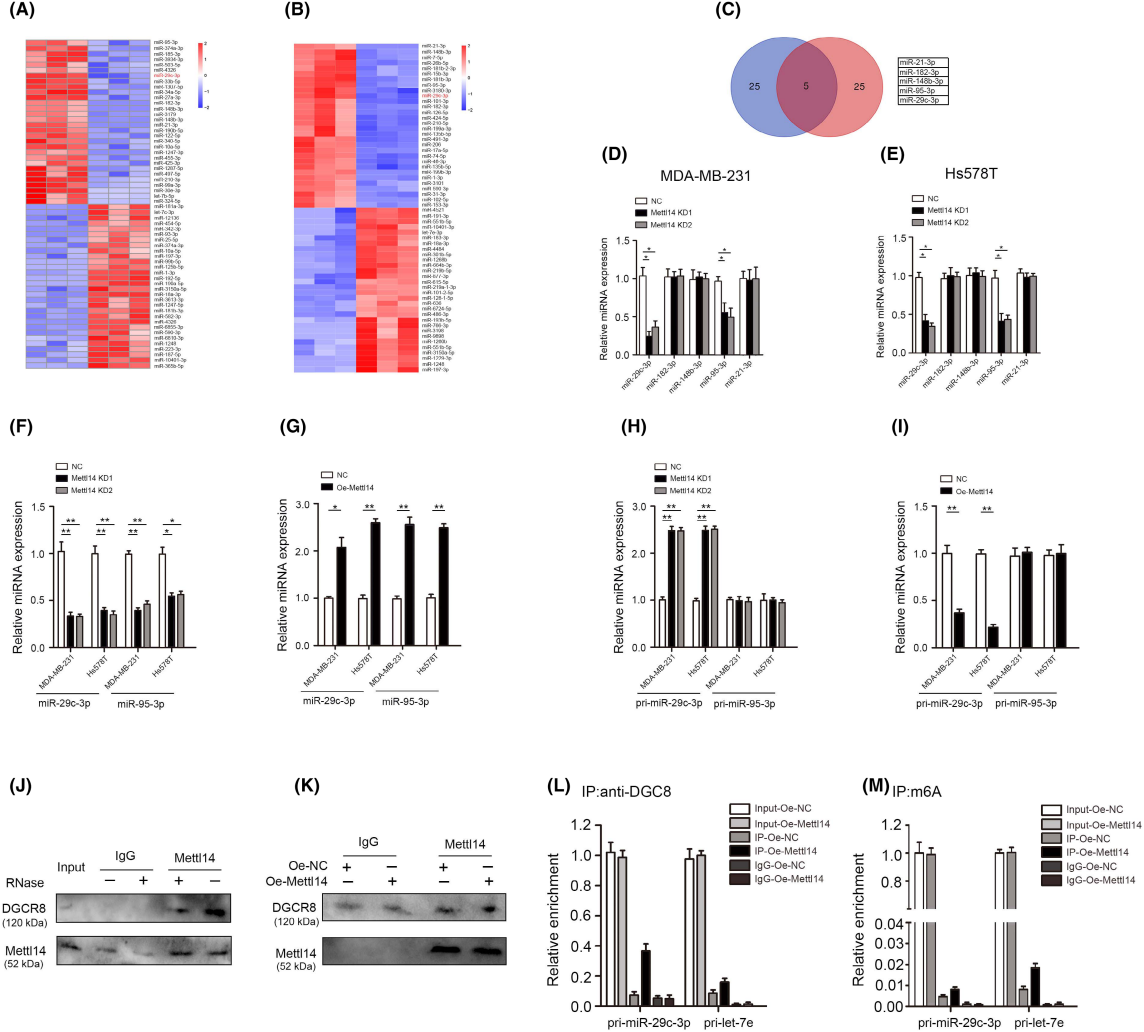
Methyltransferase‐like protein 14 (METTL14)‐dependent m6A methylation modulates the processing of miR‐29c‐3p by Di George syndrome critical region gene 8 (DGCR8). (A) A heat map showed that the differentially expressed miRNA between METTL14 knockdown group and the control group in MDA‐MB‐231 cells. (B) A heat map showed that the differentially expressed miRNA between METTL14 knockdown group and the control group in Hs578T cells. (C) Venn diagram displaying miRNAs after intersection. (D) The expression of miR‐21‐3p, miR‐182‐3p, miR‐148b‐3p, miR‐95‐3p and miR‐29c‐3p was determined by RT‐qPCR in MDA‐MB‐231 cells after METTL14 knockdown. (E) The expression of miR‐21‐3p, miR‐182‐3p, miR‐148b‐3p, miR‐95‐3p and miR‐29c‐3p was determined by RT‐qPCR in Hs578T cells after METTL14 knockdown. (F) The expression of miR‐95‐3p and miR‐29c‐3p was determined by RT‐qPCR in MDA‐MB‐231 and Hs578T cells after METTL14 knockdown. (G) The expression of miR‐95‐3p and miR‐29c‐3p was determined by RT‐qPCR in MDA‐MB‐231 and Hs578T cells after METTL14 upregulation. (H) The expression of pri‐miR‐95‐3p and pri‐miR‐29c‐3p was determined by RT‐qPCR in MDA‐MB‐231 and Hs578T cells after METTL14 knockdown. (I) The expression of pri‐miR‐95‐3p and pri‐miR‐29c‐3p was determined by RT‐qPCR in MDA‐MB‐231 and Hs578T cells after METTL14 upregulation. (J) Co‐immunoprecipitation (IP) of the METTL14‐interacting protein DGCR8. (K) IP of DGCR8, METTL14 and associated RNA from control or METTL14‐overexpressing. (L) RT‐qPCR analysis of pri‐miR‐29c‐3p binding to DGCR8 in IP assay of DGCR8‐associated RNA from control and METTL14‐overexpressing (M) RT‐qPCR analysis of the pri‐miR‐29c‐3p m6A modification level determined by IP of m6A‐modified miRNA in control or METTL14‐overexpressing. *p < 0.05, **p < 0.01.

A large number of studies has shown that METTL14 regulates pri‐miRNA generation through m6A methylation dependent on DGCR8.^32^ In order to find the specific mechanism by which METTL14 regulates miRNA through m6A methylation, we preformed transcriptome sequencing in MDA‐MB‐231 and Hs578T cells after reducing METTL14 expression (Figure 4A,B). Five miRNAs with reduced expression (miR‐21‐3p, miR‐182‐3p, miR‐148b‐3p, miR‐95‐3p and miR‐29c‐3p) were identified through intersection analysis using the Wayne plot (Figure 4C). Then, RT‐qPCR analyses suggested that miR‐29c‐3p and miR‐95‐3p were significantly downregulated in METTL14‐KD MDA‐MB‐231 and Hs578T cells (Figure 4D,E). Next, we examined the expression of miR‐29c‐3p and miR‐95‐3p in METTL14‐KD or overexpressing MDA‐MB‐231 and Hs578T cells. The results indicate that mature miR‐29c‐3p and miR‐95‐3p was downregulated in METTL14‐KD MDA‐MB‐231 and Hs578T cells, and upregulated in METTL14‐overexpressing MDA‐MB‐231 and Hs578T cells (Figure 4F,G). In addition, unprocessed pri‐miR‐29c‐3p was found to accumulate in METTL14‐KD MDA‐MB‐231 and Hs578T cells and accelerate in METTL14‐overexpressing MDA‐MB‐231 and Hs578T cells, but the KD and overexpression of METTL14 had no effect on pri‐ miR‐95‐3p expression, unlike pri‐miR‐29c‐3p (Figure 4H,I). More important, immunoprecipitation assay revealed that METTL14 co‐precipitates with DGCR8, and ribonuclease treatment weakens their interaction, suggesting that METTL14 and DGCR8 interaction might be partly mediated by RNAs (Figure 4J,K). In addition, immunoprecipitated DGCR8 from control vector and Oe‐METTL14 groups and examine pri‐miRNAs bound to DGCR8, and found that the expression of pri‐miR‐29c‐3p bound to DGCR8 was increased in Oe‐METTL14 cells (Figure. 4L). Moreover, we discovered that the expression of pri‐miR‐29c‐3p modified by m6A was increased in Oe‐METTL14 cells when m6A was immunoprecipitated from RNAs of control vector and Oe‐METTL14 groups (Figure 4M).

### 3.5 miR‐29c‐3p ameliorates the inhibitory effect of METTL14 on TNBC progression

**FIGURE 5:**
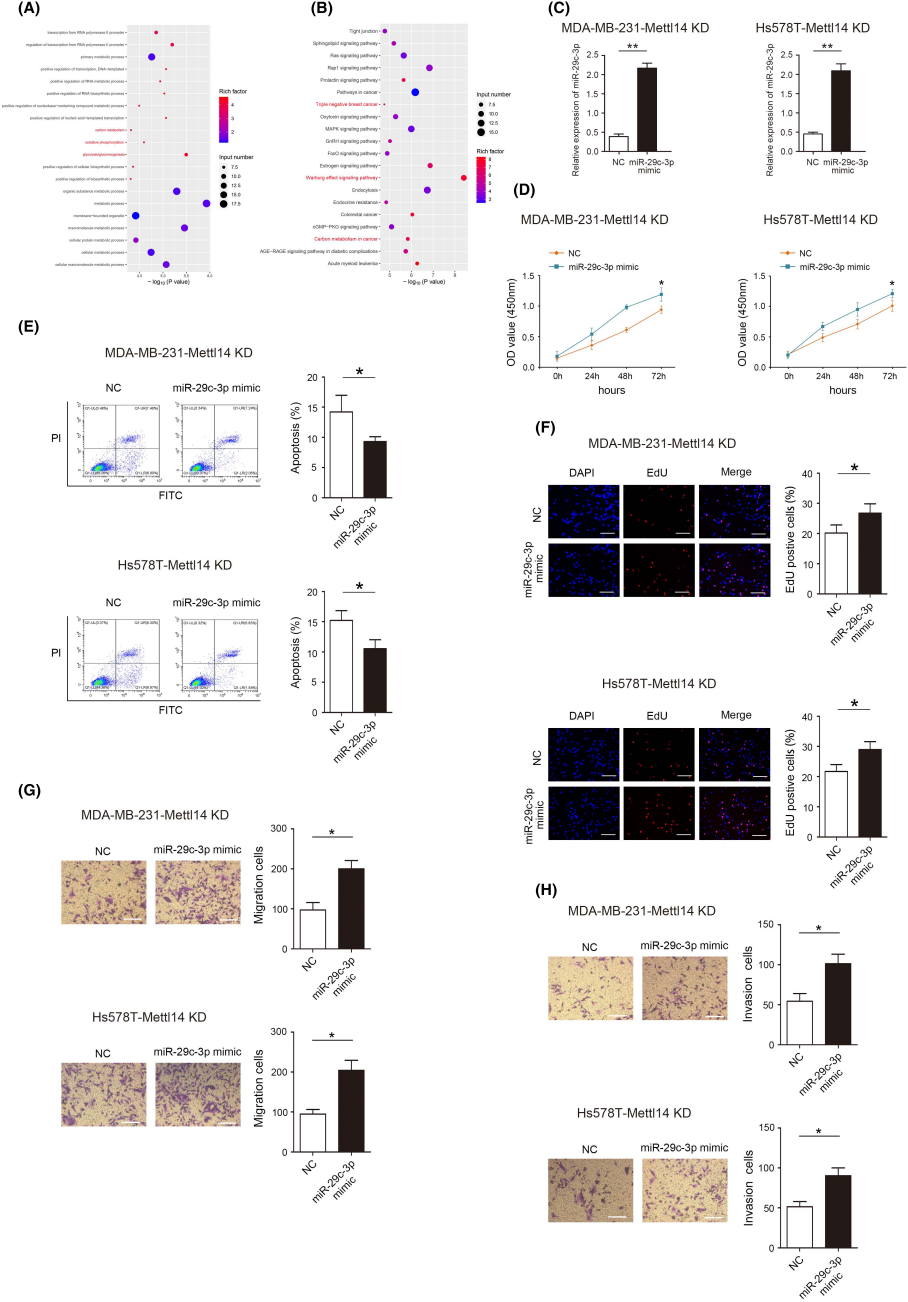
miR‐29c‐3p ameliorates the inhibitory effect of methyltransferase‐like protein 14 (METTL14) on triple‐negative breast cancer (TNBC) progression. (A, B) KEGG functional enrichment analysis showed that the differentially expressed miRNAs were enriched in TNBC, carbon metabolism and Warburg effect. (C) The expression of miR‐29c‐3p in MD‐MBA‐231‐METTL14 KD and Hs578T‐METTL14 KD cells detected by RT‐qPCR. (D) Effect of miR‐29c‐3p on MD‐MBA‐231‐METTL14 KD and Hs578T‐METTL14 KD cells proliferation detected by CCK‐8 assay. (E) Effect of miR‐29c‐3p on MD‐MBA‐231‐METTL14 KD and Hs578T‐METTL14 KD cells apoptosis detected by Flow cytometry. (F) Effect of miR‐29c‐3p on MD‐MBA‐231‐METTL14 KD and Hs578T‐METTL14 KD cells proliferation detected by EdU uptake assay. (G) Effect of miR‐29c‐3p on MD‐MBA‐231‐METTL14 KD and Hs578T‐METTL14 KD cell migration detected by transwell assays. (H) Effect of miR‐29c‐3p on MD‐MBA‐231‐METTL14 KD and Hs578T‐METTL14 KD cell invasion detected by transwell assays. *p < 0.05, **p < 0.01. Scale bars, 50 μm.

KEGG enrichment analysis revealed that miRNA after METTL14 knockdown was closely related to TNBC, carbon metabolism and Warburg effect (Figure 5A,B). First, we detected the expression of miR‐29c‐3p in BC and TNBC, and the results showed that miR‐29c‐3p was highly expressed in BC and TNBC (Figure S1). In addition, effectively reducing the expression of miR‐29c‐3p can significantly inhibit the proliferation, invasion and migration of TNBC cells, reduce the size of tumour growth and inhibit the expression of Ki‐67 in vivo (Figures S2 and S3).

Next, rescue experiments were performed to confirm whether METTL14 executed its functional effects by miR‐29c‐3p. miR‐29c‐3p expression was restored after METTL14 knockdown in MDA‐MB‐231 and Hs578T cells (Figure 5C). Restoration of miR‐29c‐3p expression significantly abolished the inhibitory effects of METTL14 KD on proliferation, migration and invasion (Figure 5D,F–H). In addition, restoration of miR‐29c‐3p expression significantly abolished the promoting effect of METTL14 KD on apoptosis rate of MDA‐MB‐231 (14.26% vs. 9.29%) and Hs578T (15.27% vs. 8.67%) (Figure 5E).

### 3.6 miR‐29c‐3p ameliorates the inhibitory effect of METTL14 on TNBC glucose metabolic reprogramming

KEGG enrichment analysis revealed METTL14 and downstream target miRNAs are involved in carbon metabolism and Warburg effect in TNBC. Therefore, we first examined the effects of METTL14 on energy metabolism and Warburg effect in TNBC. We performed an extracellular acidification rate (ECAR) assay, which reflect the cancer cell's state of overall glycolytic flux in vitro, and results suggested that reducing the expression of miR‐29c‐3p conspicuously reduced the extracellular acidification rate during the stage of glycolysis after glucose feeding and oligomycin feeding in MDA‐MB‐231and Hs578T cells (Figure S4A,F). The quantitative analysis revealed that the level of glycolysis and glycolytic capacity were both significantly reduced in miR‐29c‐3p low expression MDA‐MB‐231 and Hs578T cells (Figure S4B,G). To explore whether miR‐29c‐3p interferes the TCA cycle, the oxygen consumption rate (OCR) assay was performed to explore effect of miR‐29c‐3p on the mitochondrial oxidative respiration, and results showed that reducing the expression of miR‐29c‐3p significantly reduced the oxygen consumption rate during mitochondrial oxidative respiration stage in MDA‐MB‐231 and Hs578T cells (Figure S4C,H). The quantitative analysis revealed that the level of basal respiration and maximal respiration were both dramatically reduced in miR‐29c‐3p low expression MDA‐MB‐231 and Hs578T cells (Figure S4D,I). In addition, the levels of glucose uptake, pyruvate, lactate and the production of ATP were all significantly decreased in miR‐29c‐3p low expression in MDA‐MB‐231 and Hs578T cells (Figure S4E,J).

**FIGURE 6:**
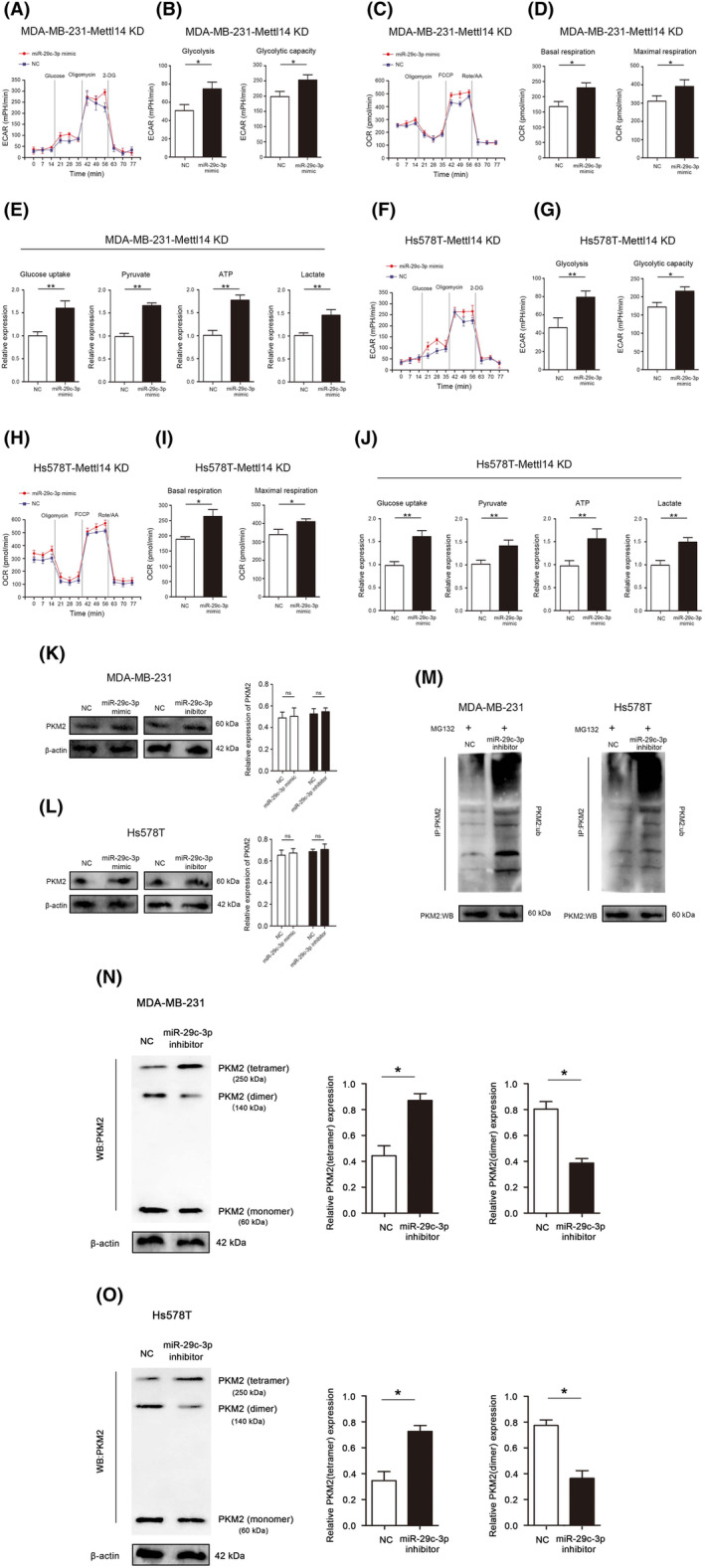
miR‐29c‐3p ameliorates the inhibitory effect of methyltransferase‐like protein 14 (METTL14) on glucose metabolic reprogramming and participates in ubiquitination regulation of PKN2 tetramer and dimer formation in triple‐negative breast cancer (TNBC). (A) ECAR assay was used to analyse overall glycolic flux in MD‐MBA‐231‐METTL14 KD cells and (F) Hs578T‐METTL14 KD cells. (B) Statistical analysis of glycolysis and glycolytic capacity in MD‐MBA‐231‐METTL14 KD cells and (G) Hs578T‐METTL14 KD cells. (C) OCR assay was used to analyse mitochondrial oxidative respiration in MD‐MBA‐231‐METTL14 KD cells and (H) Hs578T‐METTL14 KD cells. (D) Statistical analysis of basal respiration and maximal respiration in MD‐MBA‐231‐METTL14 KD cells and (I) Hs578T‐METTL14 KD cells. (E) Examination of the effects of miR‐29c‐3p overexpression on glucose uptake, pyruvate, lactate and ATP in MD‐MBA‐231‐METTL14 KD cells and (J) Hs578T‐METTL14 KD cells. (K) Protein expression of PKM2 in MD‐MBA‐231 cells after miR‐29c‐3p overexpression or knockdown. (L) Protein expression of PKM2 in Hs578T cells after miR‐29c‐3p overexpression or knockdown. (M) The levels of PKM2‐ub were examined by western blot with anti‐ubiquitin antibody in MD‐MBA‐231 and Hs578T‐METTL14 cells after reducing miR‐29c‐3p expression. (N) Western blot analysis of the protein expression levels of monomeric, dimeric and tetrameric PKM2 in MD‐MBA‐231 cells after reducing miR‐29c‐3p expression. (O) Western blot analysis of the protein expression levels of monomeric, dimeric and tetrameric PKM2 in Hs578T cells after reducing miR‐29c‐3p expression. *p < 0.05, **p < 0.01.

Next, rescue experiments were performed to confirm whether METTL14 executed its functional effects by miR‐29c‐3p. Restoration of miR‐29c‐3p expression significantly abolished the inhibitory effects of METTL14 KD on ECAR and OCR (Figure 6A–D and F–I). In addition, restoration of miR‐29c‐3p expression significantly abolished the inhibitory effects of METTL14 KD on the levels of glucose uptake, pyruvate, lactate and the production of ATP (Figure 6E,J).

### 3.7 MiR‐29c‐3p participates in ubiquitination regulation of PKM2 tetramer and dimer formation in TNBC

PKM2 is a key protein of the Warburg effect and widely involved in the glycolysis process of tumour cells.^33^, ^34^ The above‐mentioned results of the present study indicated that miR‐29c‐3p significantly regulates the glycolysis process in TNBC. Therefore, we examined whether miR‐29c‐3p has a regulatory effect on PKM2. Western blot analyses results indicated that no changes in the protein of PKM2 were observed after changing the expression of miR‐29c‐3p (Figure 6K,L).

Our previous studies have shown that PKM2 can be modified by ubiquitination and alter the protein spatial outcomes of its tetramer and dimer.^35^ The ubiquitination detection results indicated that the ubiquitination levels of PKM2 were enhanced in MDA‐MB‐231 and Hs578T cells with miR‐29c‐3p low expression (Figure 6M). Importantly, reducing expression of miR‐29c‐3p significantly increased the expression of PKM2 tetramer, decreased the expression of PKM2 dimer and increased the ratio of PKM2 tetramer in MDA‐MB‐231 and Hs578T cells (Figure 6N,O).

### 3.8 MiR‐29c‐3p directly interacts with TRIM9

**FIGURE 7:**
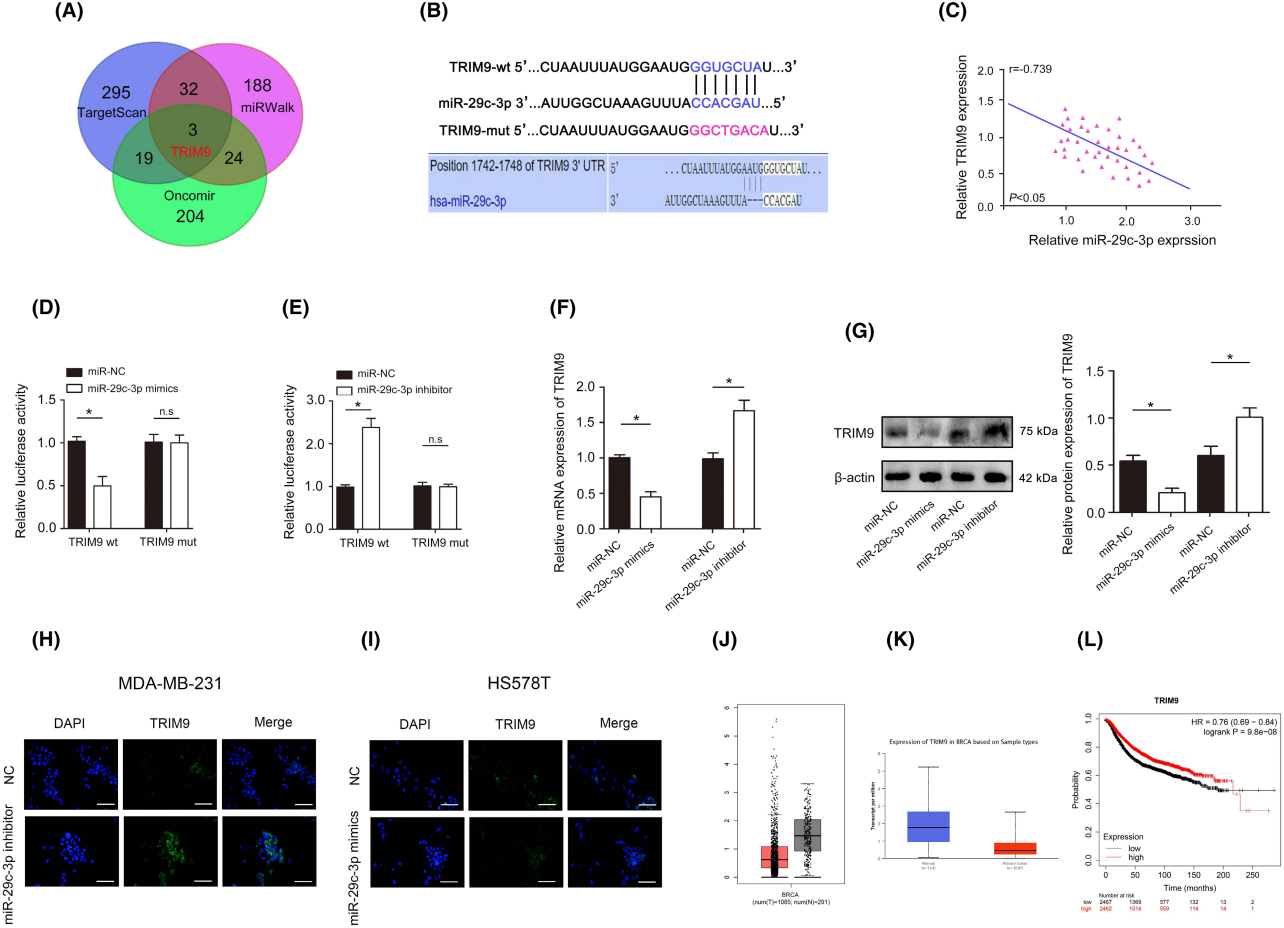
miR‐29c‐3p directly targets TRIM9. (A) Venn diagram displaying miR‐29c‐3p computationally predicted to target TRIM9 by TargetScan, miRanda and miRWalk. (B) Diagrams reveal putative miR‐29c‐3p binding sites and corresponding mutant sites of TRIM9. (C) miR‐29c‐3p expression was negatively correlated with TRIM9 expression in triple‐negative breast cancer (TNBC). (D) The luciferase activity of each combination was assessed in miR‐29c‐3p overexpression. (E) The luciferase activity of each combination was assessed in miR‐29c‐3p knockdown. (F) TRIM9 mRNA expression in miR‐29c‐3p overexpression or knockdown. (G) TRIM9 protein expression in miR‐29c‐3p overexpression or knockdown. (H) TRIM9 protein expression in MDA‐MB‐231 cells after miR‐29c‐3p knockdown was detected by immunofluorescence. (I) TRIM9 protein expression in Hs578T cells after miR‐29c‐3p overexpression was detected by immunofluorescence. (J) mRNA expression of TRIM9 in TCGA database. (K) mRNA expression of TRIM9 in the GEPIA database. (L) Kaplan–Meier curves indicating the relationship between TRIM9 expression and the prognosis of patients with breast cancer (BC) in TCGA database. *p < 0.05. Scale bars, 50 μm.

Increasing studies have shown that miRNA affects the biological behaviour of tumour cells by regulating the expression of downstream target genes.^36^ We examined miRNA databases, including TargetScan, miRWalk and Oncomir, and identified that TRIM9 was listed as a potential target of miR‐29c‐3p (Figure 7A). Complementary sequence of miR‐29c‐3p was found in the 3’‐UTR of TRIM9 mRNA (Figure 7B). Statistical analysis shows that miR‐29c‐3p expression was inversely associated with TRIM9 (Figure 7C). Next, co‐transfection of miR‐29c‐3p significantly inhibited luciferase activity in cells transfected with Wt TRIM9 3’‐UTR. However, the inhibition was not observed in cells co‐transfected with Mt TRIM9 3’‐UTR (Figure 7D,E). As shown in Figure 7F,G, the results revealed that miR‐29c‐3p overexpression significantly reduced TRIM9 mRNA and protein levels, and reducing expression of miR‐29c‐3p significantly increased TRIM9 mRNA and protein levels. In addition, the results of cellular immunofluorescence also indicated that miR‐29c‐3p overexpression significantly reduced TRIM9 expression, and reducing expression of miR‐29c‐3p significantly increased TRIM9 expression in MDA‐MB‐231 and Hs578T cells (Figure 7H,I). The analysis of bioinformatics database data indicated that TRIM9 is low expressed in BC, and its low expression indicates poor prognosis for patients (Figure 7J–L).

### 3.9 TRIM9 ameliorates the inhibitory effect of miR‐29c‐3p on TNBC progression

**FIGURE 8:**
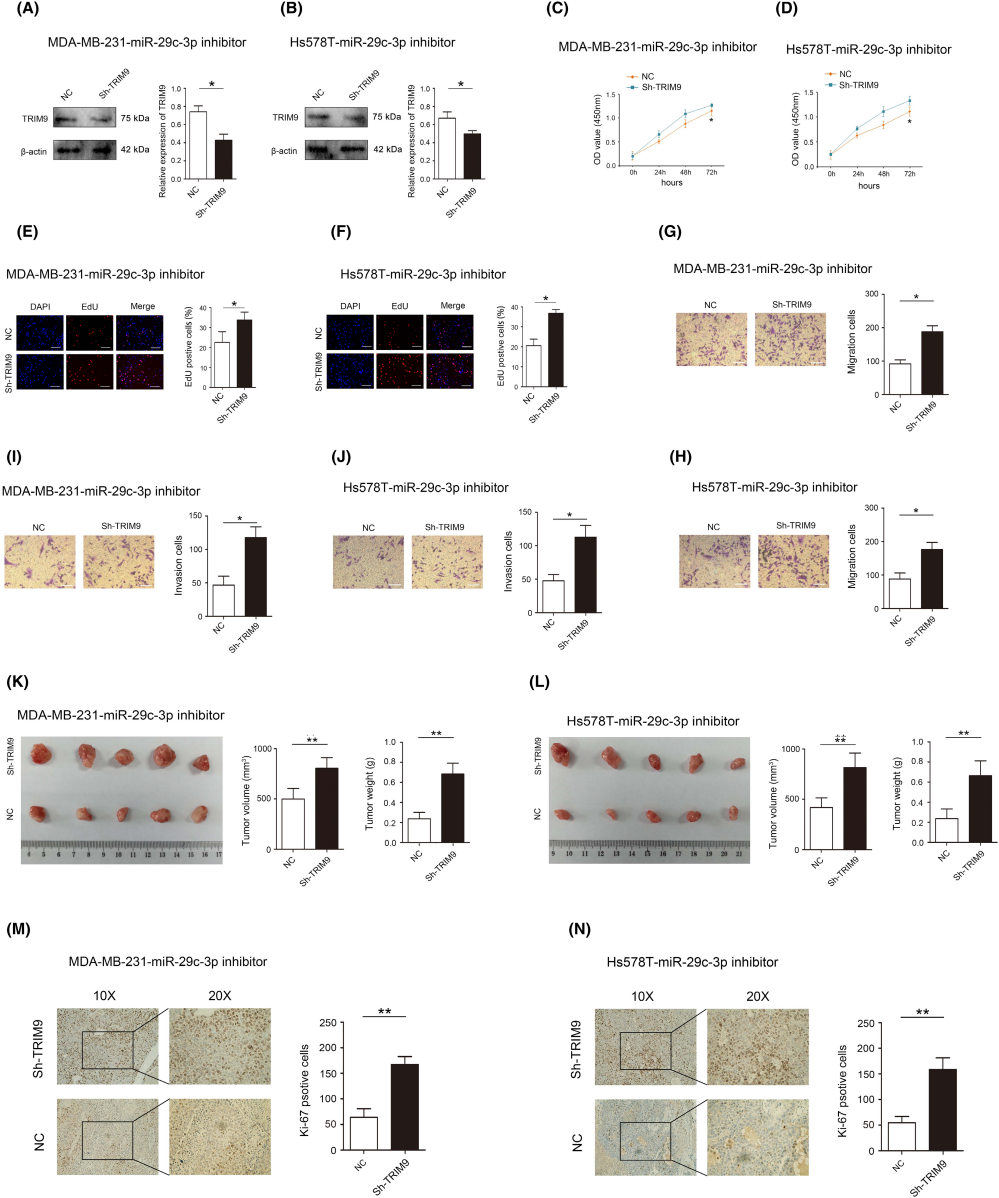
TRIM9 ameliorates the inhibitory effect of miR‐29c‐3p on triple‐negative breast cancer (TNBC) progression. (A) The expression of TRIM9 in MDA‐MB‐231‐miR‐29c‐3p inhibitor cells detected by western blot. (B) The expression of TRIM9 in Hs578T‐miR‐29c‐3p inhibitor cells detected by western blot. (C) Effect of TRIM9 on MDA‐MB‐231‐miR‐29c‐3p inhibitor cells proliferation detected by CCK‐8 assay. (D) Effect of TRIM9 on Hs578T‐miR‐29c‐3p inhibitor cells proliferation detected by CCK‐8 assay. (E) Effect of TRIM9 on MDA‐MB‐231‐miR‐29c‐3p inhibitor cell proliferation detected by EdU uptake assay. (F) Effect of TRIM9 on Hs578T‐miR‐29c‐3p inhibitor cell proliferation detected by EdU uptake assay. (G) Effect of TRIM9 on MDA‐MB‐231‐miR‐29c‐3p inhibitor cell migration detected by transwell assays. (H) Effect of TRIM9 on Hs578T‐miR‐29c‐3p inhibitor cell migration detected by transwell assays. (I) Effect of TRIM9 on MDA‐MB‐231‐miR‐29c‐3p inhibitor cell invasion detected by transwell assays. (J) Effect of TRIM9 on Hs578T‐miR‐29c‐3p inhibitor cell invasion detected by transwell assays. (K) Effect of TRIM9 on MDA‐MB‐231‐miR‐29c‐3p inhibitor cell proliferation detected by nude mouse tumour model. (L) Effect of TRIM9 on Hs578T‐miR‐29c‐3p inhibitor cell proliferation detected by nude mouse tumour model. (M) Effect of TRIM9 on Ki‐67 expression in MDA‐MB‐231‐miR‐29c‐3p inhibitor cells detected by immunohistochemistry. (N) Effect of TRIM9 on Ki‐67 expression in Hs578T‐miR‐29c‐3p inhibitor cells detected by immunohistochemistry. *p < 0.05, **p < 0.01. Scale bars, 50 μm.

First, effectively increasing the expression of TRIM9 can significantly inhibit the proliferation, invasion and migration of TNBC cells (Figure S5). Next, rescue experiments were performed to confirm whether miR‐29c‐3p executed its functional effects by TRIM9. TRIM9 expression was reduced after reducing expression of miR‐29c‐3p in MDA‐MB‐231 and Hs578T cells (Figure 8A,B). Reducing of TRIM9 expression significantly abolished the inhibitory effects of miR‐29c‐3p inhibitor on proliferation, migration and invasion (Figure 8C–J). In addition, reducing of TRIM9 expression significantly abolished the inhibitory effect of miR‐29c‐3p inhibitor on the size of tumour growth and the expression of Ki‐67 in vivo (Figure 8K–N).

### 3.10 TRIM9 ameliorates the effect of miR‐29c‐3p on ubiquitination regulation of PKN2 tetramer and dimer formation in TNBC

**FIGURE 9:**
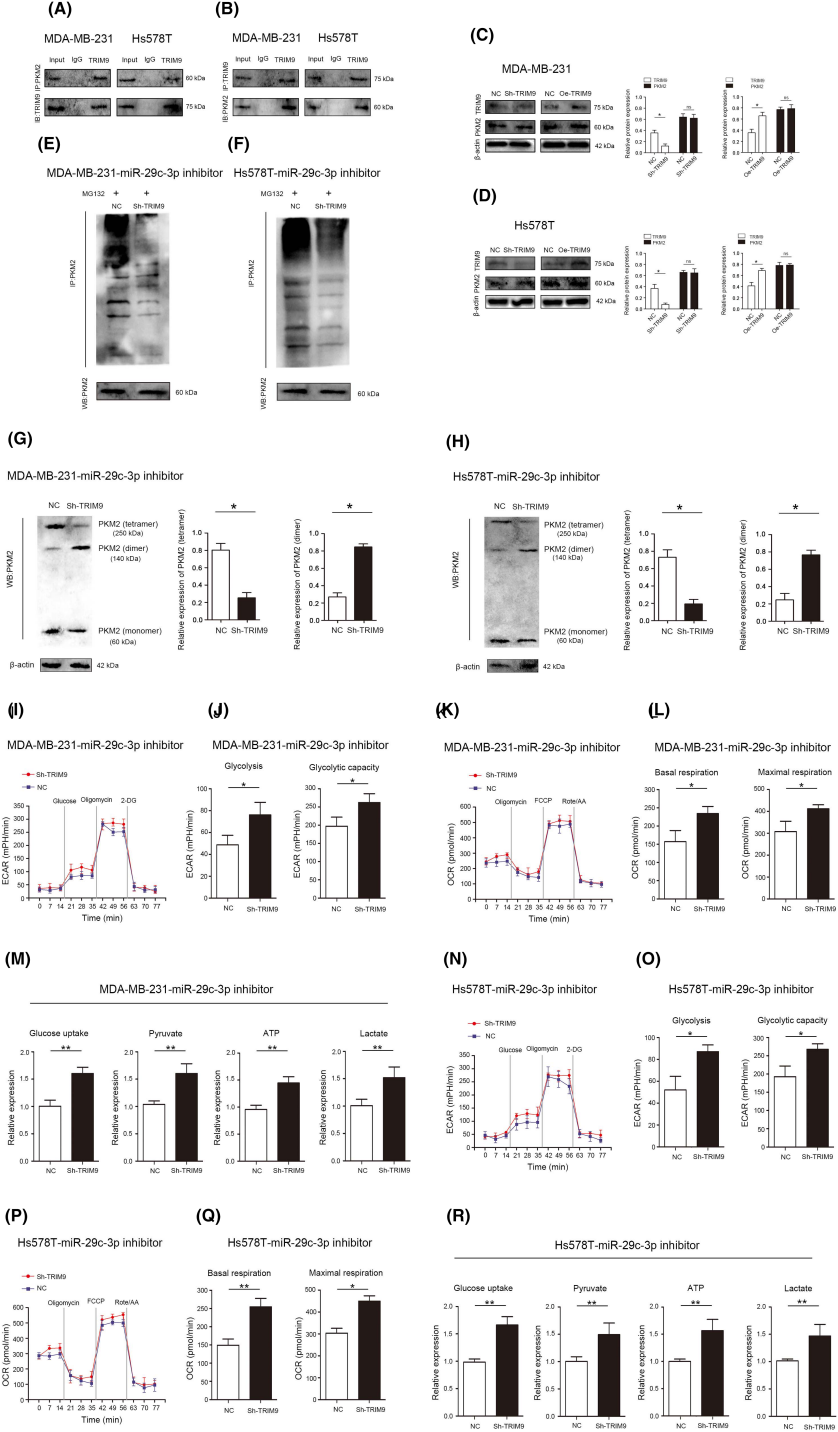
TRIM9 ameliorates the effect of miR‐29c‐3p on PKM2 and glucose metabolic reprogramming in triple‐negative breast cancer (TNBC). (A, B) Co‐IP experiments indicated that TRIM9 interacted with PKM2 in the MDA‐MB‐231 and Hs578T cells. (C) Protein expression of TRIM9 and PKM2 in MDA‐MB‐231 cells after TRIM9 overexpression or knockdown. (D) Protein expression of TRIM9 and PKM2 in Hs578T cells after TRIM9 overexpression or knockdown. (E) The levels of PKM2‐ub were examined by western blot with anti‐ubiquitin antibody in MD‐MBA‐231‐miR‐29c‐3p inhibitor cells after reducing TRIM9 expression. (F) The levels of PKM2‐ub were examined by western blot with anti‐ubiquitin antibody in Hs578T‐miR‐29c‐3p inhibitor cells after reducing TRIM9 expression. (G) Western blot analysis of the protein expression levels of monomeric, dimeric and tetrameric PKM2 in MD‐MBA‐231‐miR‐29c‐3p inhibitor cells after reducing TRIM9 expression. (H) Western blot analysis of the protein expression levels of monomeric, dimeric and tetrameric PKM2 in Hs578T‐miR‐29c‐3p inhibitor cells after reducing TRIM9 expression. (I) ECAR assay was used to analyse overall glycolic flux in MD‐MBA‐231‐miR‐29c‐3p inhibitor cells and (N) Hs578T‐miR‐29c‐3p inhibitor cells after reducing TRIM9 expression. (J) Statistical analysis of glycolysis and glycolytic capacity in MD‐MBA‐231‐miR‐29c‐3p inhibitor cells and (O) Hs578T‐miR‐29c‐3p inhibitor cells after reducing TRIM9 expression. (K) OCR assay was used to analyse mitochondrial oxidative respiration in MD‐MBA‐231‐miR‐29c‐3p inhibitor cells and (P) Hs578T‐miR‐29c‐3p inhibitor cells after reducing TRIM9 expression. (L) Statistical analysis of basal respiration and maximal respiration in MD‐MBA‐231‐miR‐29c‐3p inhibitor cells and (Q) Hs578T‐miR‐29c‐3p inhibitor cells after reducing TRIM9 expression. (M) Examination of the effects of reducing TRIM9 expression on glucose uptake, pyruvate, lactate and ATP in MD‐MBA‐231‐miR‐29c‐3p inhibitor cells and (R) Hs578T‐miR‐29c‐3p inhibitor cells after reducing TRIM9 expression. *p < 0.05, **p < 0.01.

Relationships between TRIM9 and PKM2 were verified by using reciprocal Co‐IP experiments carried out in MDA‐MB‐231 and Hs578T cells, and results confirmed the interaction between endogenous TRIM9 and PKM2 (Figure 9A,B). However, no changes in the protein of PKM2 were observed after changing the expression of TRIM9 (Figure 9C,D). Reducing expression of TRIM9 significantly abolished the promoting effects of miR‐29c‐3p inhibitor on ubiquitination of PKM2 in MDA‐MB‐231 and Hs578T cells (Figure 9E,F). More importantly, reducing expression of TRIM9 significantly abolished the promoting effects of miR‐29c‐3p inhibitor on PKM2 tetramer and inhibitory effects of miR‐29c‐3p on PKM2 dimer in MDA‐MB‐231 and Hs578T cells (Figure 9G,H).

### 3.11 TRIM9 ameliorates the inhibitory effect of miR‐29c‐3p on TNBC glucose metabolic reprogramming

**FIGURE 10:**
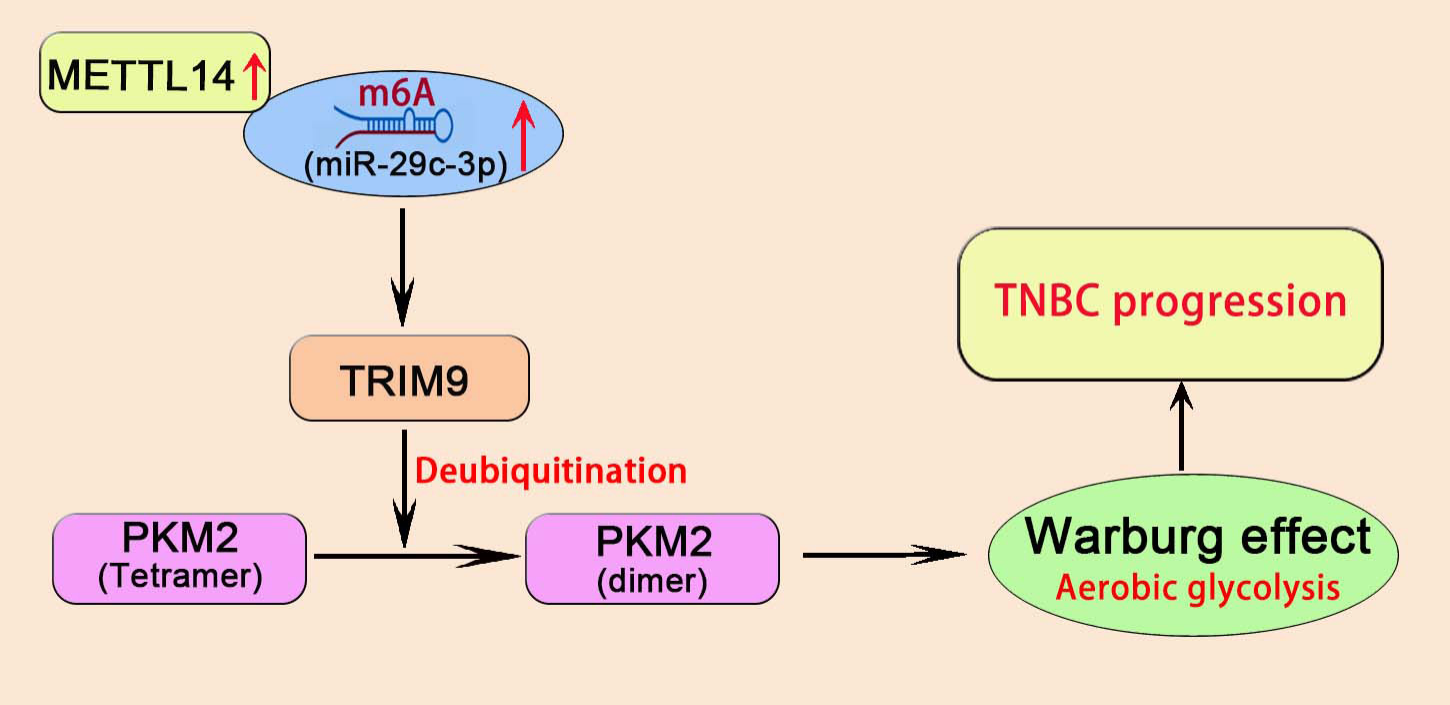
Schematic model showing important roles of methyltransferase‐like protein 14 (METTL14) in triple‐negative breast cancer (TNBC) glucose metabolic reprogramming and tumorigenesis. METTL14 activates miR‐29c‐3p through m6A methylation and regulates ubiquitination of TIRM9 to promote the conversion of PKM2 from tetramer to dimer, resulting in glucose metabolic reprogramming from oxidative phosphorylation to aerobic glycolysis to promote the progress of TNBC.

Increasing expression of TRIM9 significantly reduces ECAR, OCR, the levels of glucose uptake, pyruvate and lactate, and the production of ATP in MDA‐MB‐231 and Hs578T cells (Figure S6). In addition, rescue experiments were performed to confirm whether miR‐29c‐3p executed its functional effects by TRIM9. Reducing of TRIM9 expression significantly abolished the effects of miR‐29c‐3p inhibitor on ECAR and OCR (Figure 9I–L,N–R). Then, reducing of TRIM9 expression significantly abolished the effects of miR‐29c‐3p inhibitor on the levels of glucose uptake, pyruvate, lactate and the production of ATP (Figure 9M,R). Overall, these results demonstrated that METTL14‐mediated m6A‐induced miR‐29c‐3p promotes glucose metabolic reprogramming and TNBC progression via regulating PKM2 dimer formation through TRIM9 ubiquitination (Figure 10).

## 4 DISCUSSION

Scientists have found that m6A methylation modification plays an important role in the functioning of miRNA. METTL14, as the methyltransferase capable of binding S‐adenosylmethionine (SAM), can provide a material basis for m6A methylation modification of miRNA.^37^ m6A methylation modification will lead to the methylation of pri‐miRNA, and the pri‐miRNA that undergoes m6A methylation can be recognized and processed by DGCR8, promoting the maturation of miRNA.^38^, ^39^ The abnormal expression of METTL14 in multiple solid tumours is associated with poor prognosis.^40^ Studies have shown that low expression of METTL14 in gastric cancer reduces the m6A methylation level of circORC5 and increases the expression of circORC5.^41^ In addition, circORC5 can absorb miR‐30c‐2‐3p, reverse the upregulation of miR‐30c‐2‐3p and downregulation of proline‐rich AKT1 substrate 1 (AKT1S1) and eukaryotic initiation factor 4B (EIF4B) induced by METTL14 and regulate the malignant progression of gastric cancer cells.^41^ In papillary thyroid cancer, METTL14 regulates miR‐98 expression by inhibiting OIP5‐AS1 expression and activates epidermal growth factor receptor (EGFR), MEK/ERK pathways to inhibit proliferation and migration/invasion of papillary thyroid cancer cells.^42^ In breast cancer, METTL14 is significantly upregulated in breast cancer tumour tissue.^43^ Increased and decreased METTL14 expression regulates m6A levels in MCF‐7 and MDA‐MB‐231 cells.^43^ In addition, abnormal expression of METTL14 reshapes the miRNA map in BC cell lines. METTL14 promotes cell migration and invasion of breast cancer through miR‐146a‐5p.^43^ Our findings are consistent with previous studies that METTL14 is highly expressed in breast cancer and higher expressed in TNBC. Decreasing the expression of METTL14 can significantly inhibit the malignant biological behaviour of TNBC. Importantly, the results of Co‐IP indicate that METTL14 and DGCR8 interaction might be partly mediated by RNAs. At the same time, it was further confirmed that METTL14 regulates pri‐miR‐29c‐3p through m6A methylation modification, and positively regulates the expression of mature miR‐29c‐3p.

TRIM family proteins (TRIM) are a class of RING type ubiquitin E3 ligases that are widely involved in the ubiquitination modification of proteins and are closely related to the occurrence and development of various malignant tumours.^44^, ^45^, ^46^ TRIM9 is a member of the TRIM family of proteins and a key enzyme for ubiquitination of proteins.^47^ Research shows that TRIM9 is low expressed in breast cancer, significantly inhibits the ubiquitination level of oncogene and promotes its protein expression and function.^48^ The occurrence and development of tumours is a process of inactivation of tumour suppressor genes and uncontrolled continuous division and proliferation of tumour cells.^48^ TRIM9 is a key protein that regulates the inactivation of ubiquitination of tumour suppressor genes. In uterine fibroids, TRIM9 is overexpressed and regulates cyclin D1, survivin, lysed caspase 3 and nuclear NF‐κB to enhance cell proliferation, reduces apoptosis and significantly promotes the growth of uterine fibroids.^49^ TRIM9‐dependent ubiquitination blocks interaction and phosphorylation with FAK. Under the stimulation of netrin‐1, TRIM9 promotes the malignant proliferation of colorectal cancer cells and reduces the prognosis of patients.^50^ TRIM9 short isoforms (TRIM9s) promote ubiquitination of the K63 junction of MKK6 at lysine 82, thereby inhibiting ubiquitination of the degradable K48 junction at the same lysine location of Mitogen activated protein kinase kinase 6 (MKK6).^51^ In addition, MKK6 can also promote the phosphorylation of TRIM9s in Ser76/80 through p38, thereby blocking the ubiquitin‐proteasome pathway and stabilizing the structure of TRIM9s.^51^ Through the above mechanisms, TRIM9 can inhibit the malignant progression of glioblastoma. Importantly, our research results show that PKM2 is regulated by ubiquitination of TRIM9, but this regulation mechanism does not affect the protein expression of PKM2, but affects the protein spatial structure of PKM2, regulates the expression of tetramer of dimer of PKM2 and affects the energy metabolism process of TNBC through Warburg effect and ultimately regulates the malignant progression of TNBC.

MiRNA is a class of small single‐stranded non‐coding RNAs with a length of 21–23 nt.^52^ They are transcribed by RNA polymerase II and III to form long chain pri‐miRNAs or pre‐miRNAs, which are then treated by ribosomal enzymes to form mature bodies.^53^ These mature miRNAs are integrated into RNA‐induced silencing complex (RISC). It binds to specific sites in the 3′ noncoding region of the target mRNA in an incomplete complementary manner, thereby mediating mRNA degradation or inhibiting protein translation and plays a key role in mRNA silencing and regulation of gene post‐transcriptional expression.^54^, ^55^, ^56^ MiRNA plays an important role in the occurrence and development of breast cancer. For example, miR‐182‐3p is a specific and effective post‐transcriptional regulator of telomeric repeat binding factor 2 (TRF2).^57^ Ectopic expression of miR‐182‐3p significantly reduced TRF2 protein levels in telomerase‐positive cancer cell lines or one selective prolongation cell line.^57^ At the same time, miR‐182‐3p induced DNA damage at telomere and peritelomere sites, eventually leading to strong apoptosis activation and promoting malignant progression of breast cancer.^57^ miR‐142‐3p was found to alter paclitaxel resistance by targeting recombinant G protein beta 2 (GNB2), regulate migration and autophagy energy of breast cancer cells, and further reveal that knockdown of GNB2 expression can activate AKT–mTOR pathway.^58^ Clinically, the high expression of miR‐1275 in plasma indicates a better response to neoadjuvant chemotherapy in breast cancer. The reduction of miR‐1275 increases the ratio of cancer stem cells by targeting MDK/AKT axis, thus promoting the chemotherapy resistance of breast cancer cells.^59^ In our study, co‐transfection of miR‐29c‐3p significantly inhibited luciferase activity in cells transfected with Wt TRIM9 3’‐UTR, which suggested that miRNA plays a role by binding the regulatory mechanism of target gene mRNA sequence through classical miRNA. The results of WB and RT‐qPCR indicate that miR‐29c‐3p can significantly regulate the expression of TRIM9 and affect its ubiquitination regulation and play a significant regulatory role in tetramer and dimer formation of PKM2.

Collectively, the results of the present study indicated that METTL14 may regulate the expression of miR‐29c‐3p through m6A methylation, which further regulates the ubiquitination modification ability of TRIM9 and affects the formation of dimers and tetramers of PKM2, which affects the malignant biological behaviour and glucose metabolic reprogramming from oxidative phosphorylation to aerobic glycolysis of TNBC via Warburg effect.

## AUTHOR CONTRIBUTIONS

**Hao Wu:** Conceptualization (equal); data curation (equal); formal analysis (equal); funding acquisition (equal); investigation (equal); methodology (equal); project administration (equal); writing – original draft (equal); writing – review and editing (equal). **Yile Jiao:** Conceptualization (equal); data curation (equal); resources (equal); software (equal); supervision (equal); writing – original draft (equal). **Xinyi Guo:** Project administration (equal); resources (equal); software (equal); supervision (equal); validation (equal). **Zhenru Wu:** Methodology (equal); project administration (equal); resources (equal); software (equal); supervision (equal); validation (equal). **Qing Lv:** Conceptualization (equal); data curation (equal); formal analysis (equal); funding acquisition (equal).

## FUNDING INFORMATION

This work is supported by the National Natural Science Foundation of China (82100655), the Department of Science and Technology of Sichuan province, China (2022YFS0313), the Department of Science and Technology of Sichuan province, China (2022YFQ0003).

## CONFLICT OF INTEREST STATEMENT

The authors declare that they have no competing interests.

## CONSENT FOR PUBLICATION

All authors consent to publication.

## Supporting information


Figure S1.

Figure S2.

Figure S3.

Figure S4.

Figure S5.

Figure S6.

Table S1.

## Data Availability

The data that support the findings of this study are available on request from the corresponding author. The data are not publicly available due to privacy or ethical restrictions.
