# Supplementary figures and images for: METTL14/miR‐29c‐3p axis drives aerobic glycolysis to promote triple‐negative breast cancer progression though TRIM9‐mediated PKM2 ubiquitination
Source: J Cell Mol Med. 2024 Jan 23;28(3):e18112. doi: 10.1111/jcmm.18112 (PMC10844685; doi:10.1111/jcmm.18112)

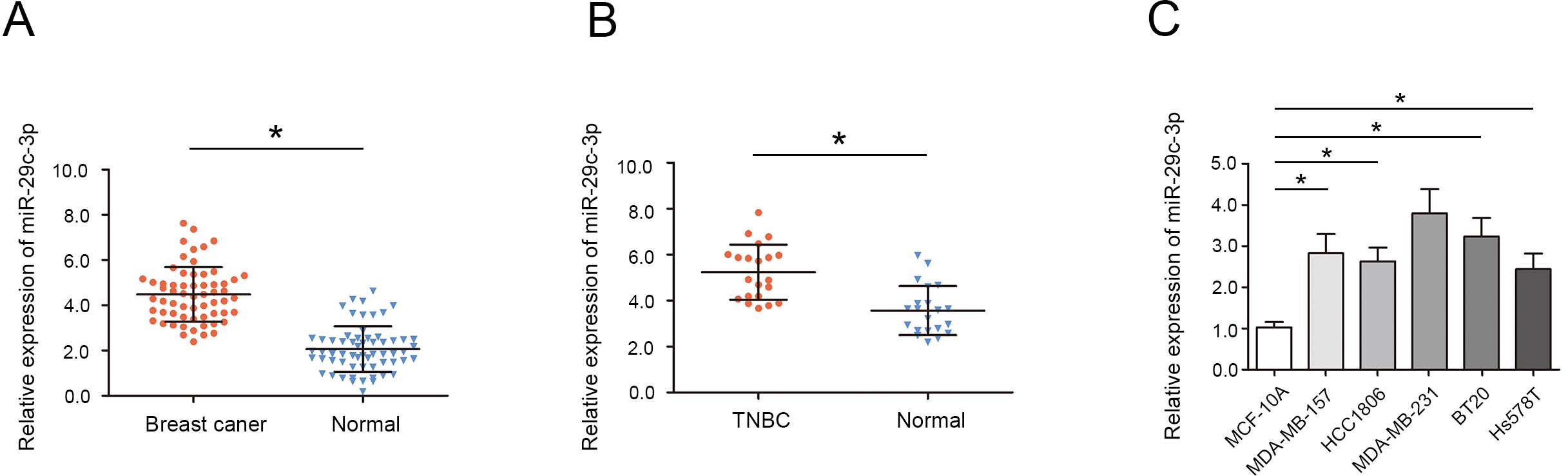

Supplement: Supplementary file 1 — Figure S1. [file JCMM-28-e18112-s004.jpg]

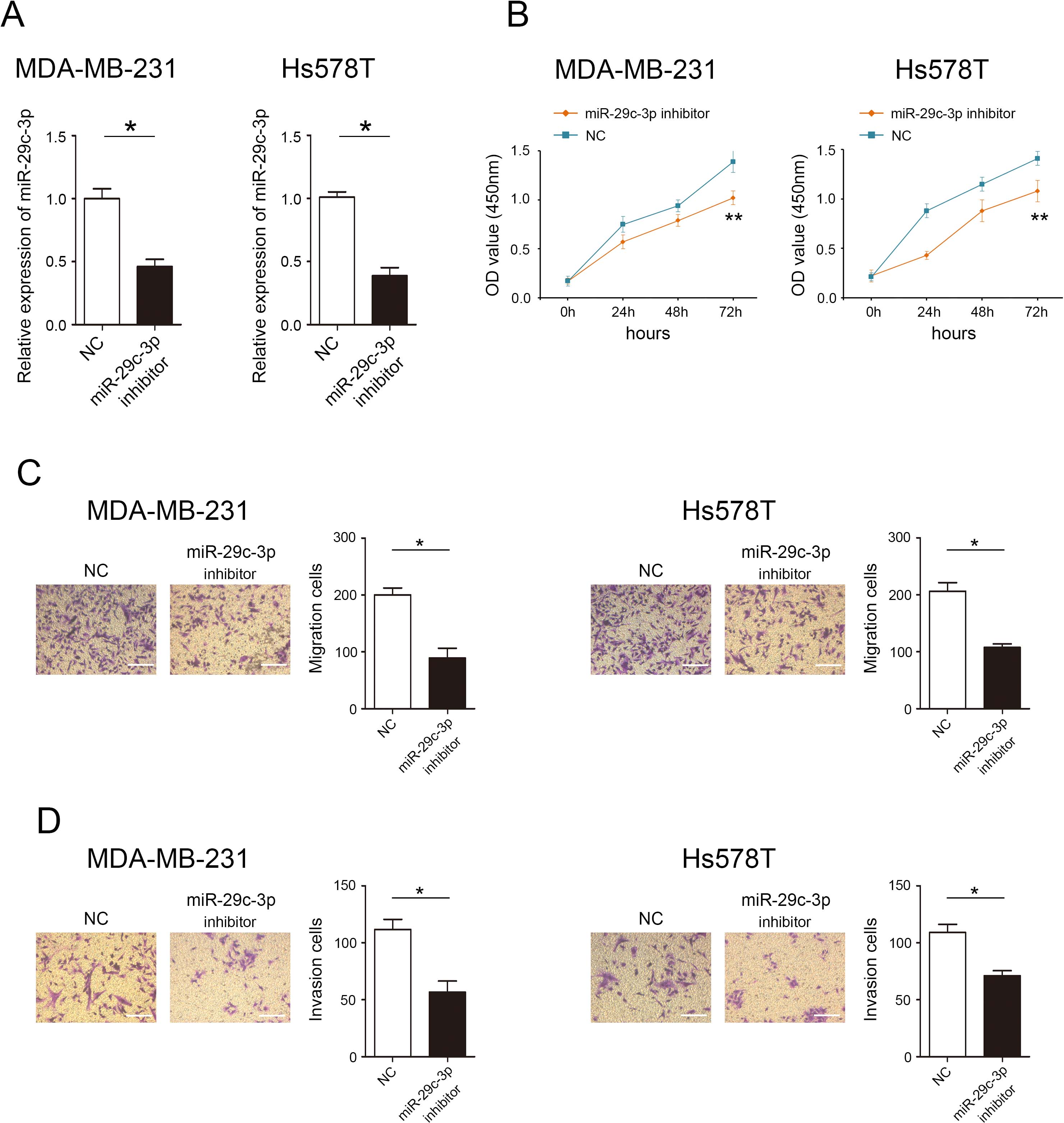

Supplement: Supplementary file 2 — Figure S2. [file JCMM-28-e18112-s003.jpg]

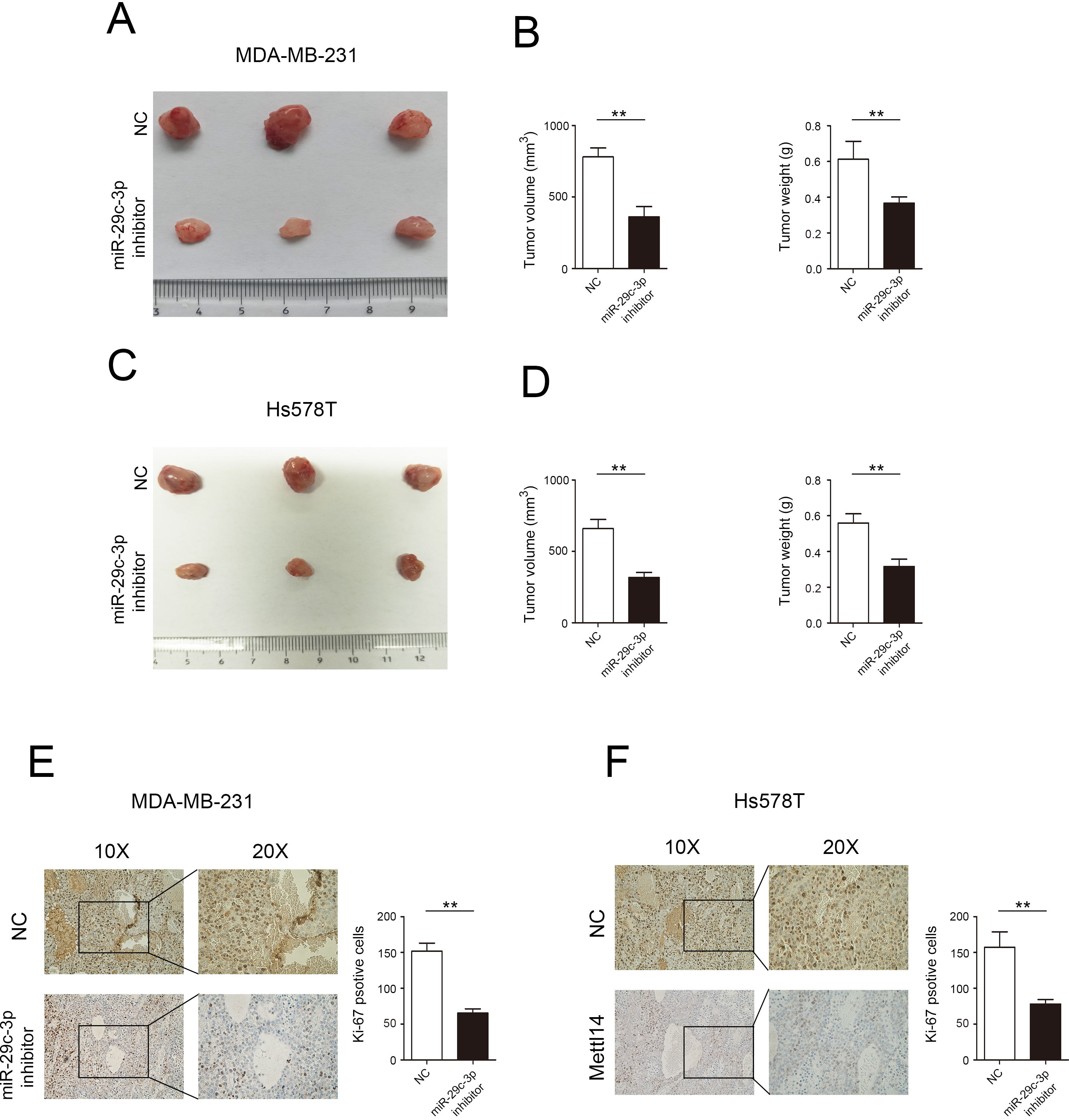

Supplement: Supplementary file 3 — Figure S3. [file JCMM-28-e18112-s005.jpg]

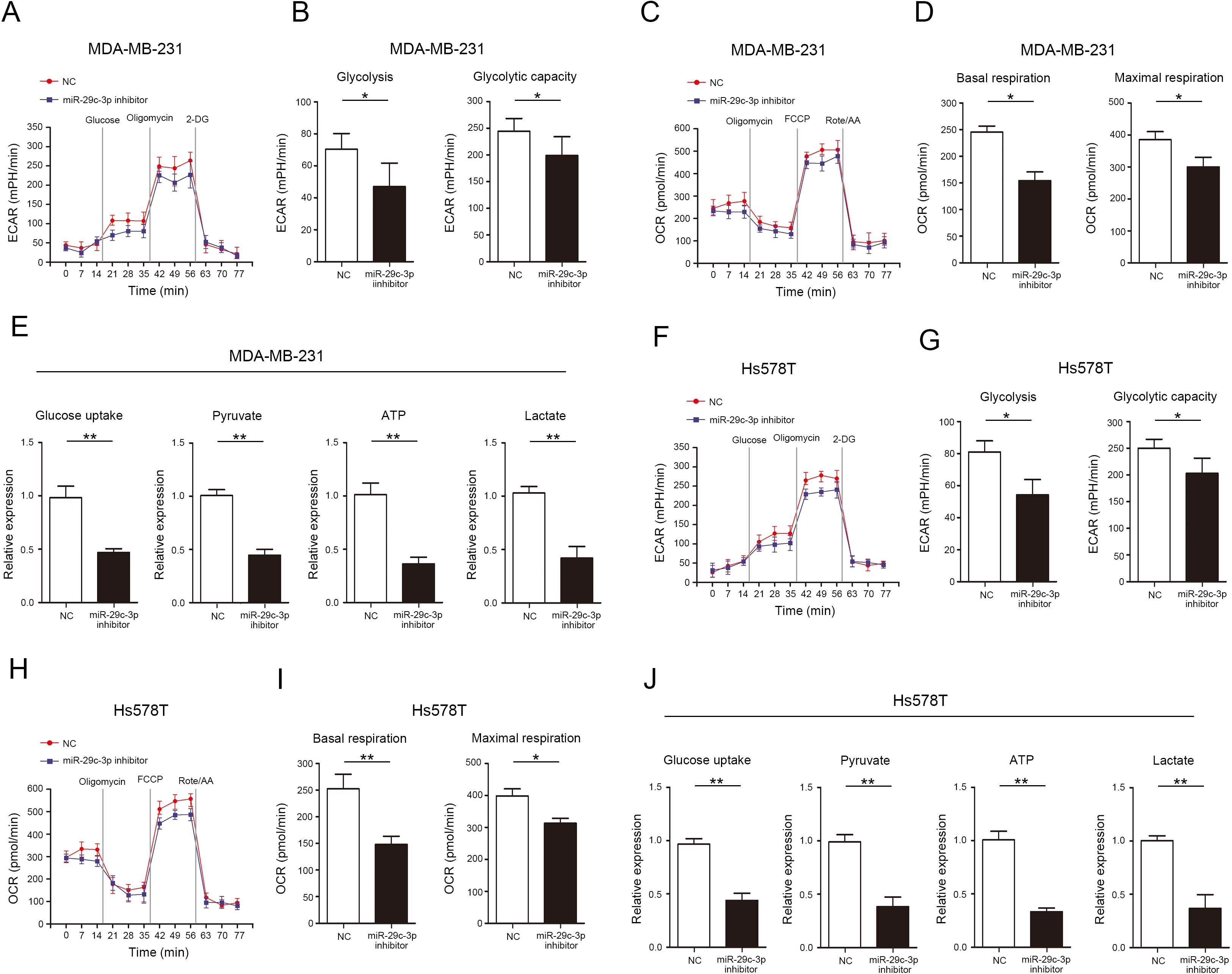

Supplement: Supplementary file 4 — Figure S4. [file JCMM-28-e18112-s001.jpg]

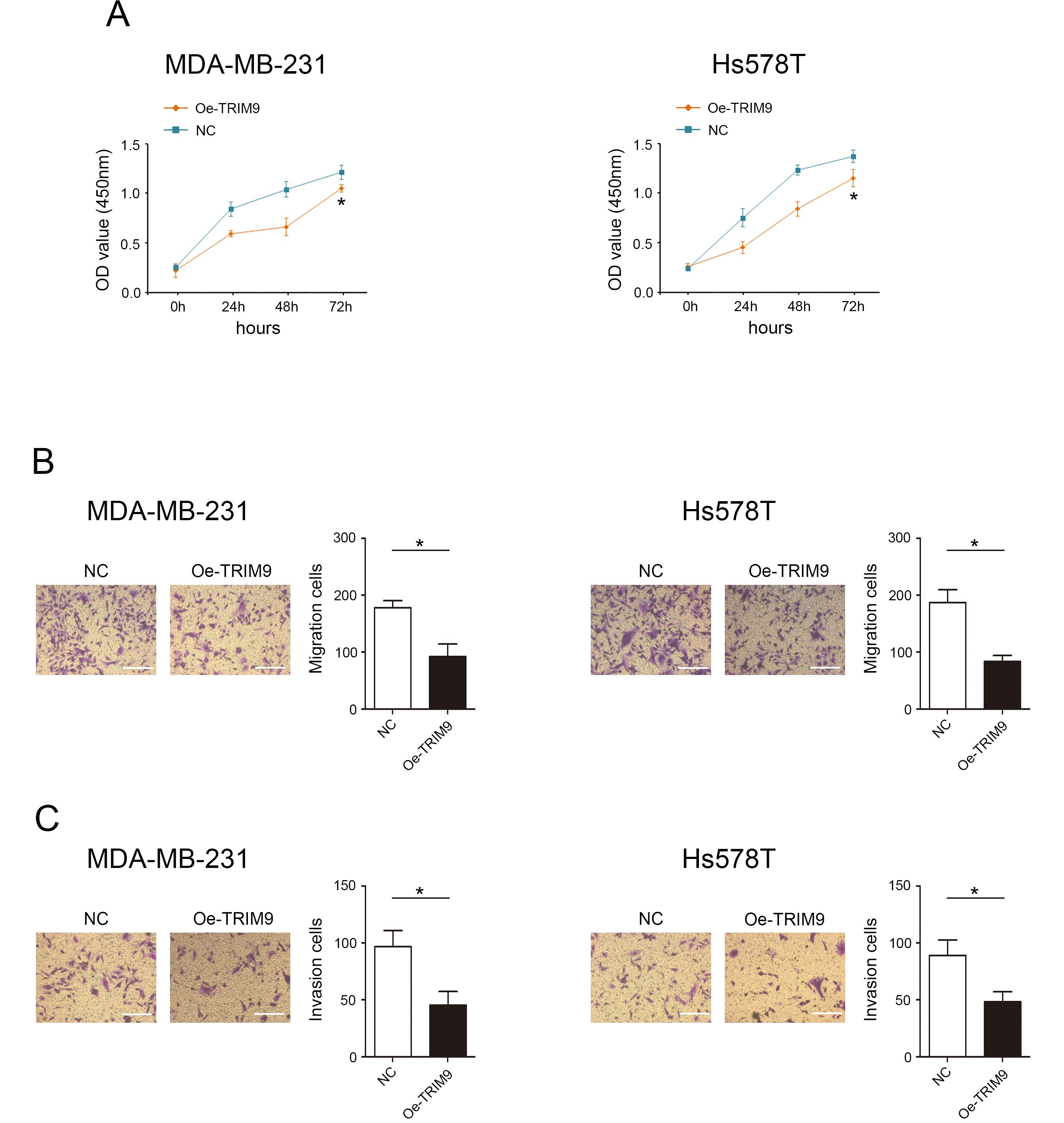

Supplement: Supplementary file 5 — Figure S5. [file JCMM-28-e18112-s007.jpg]

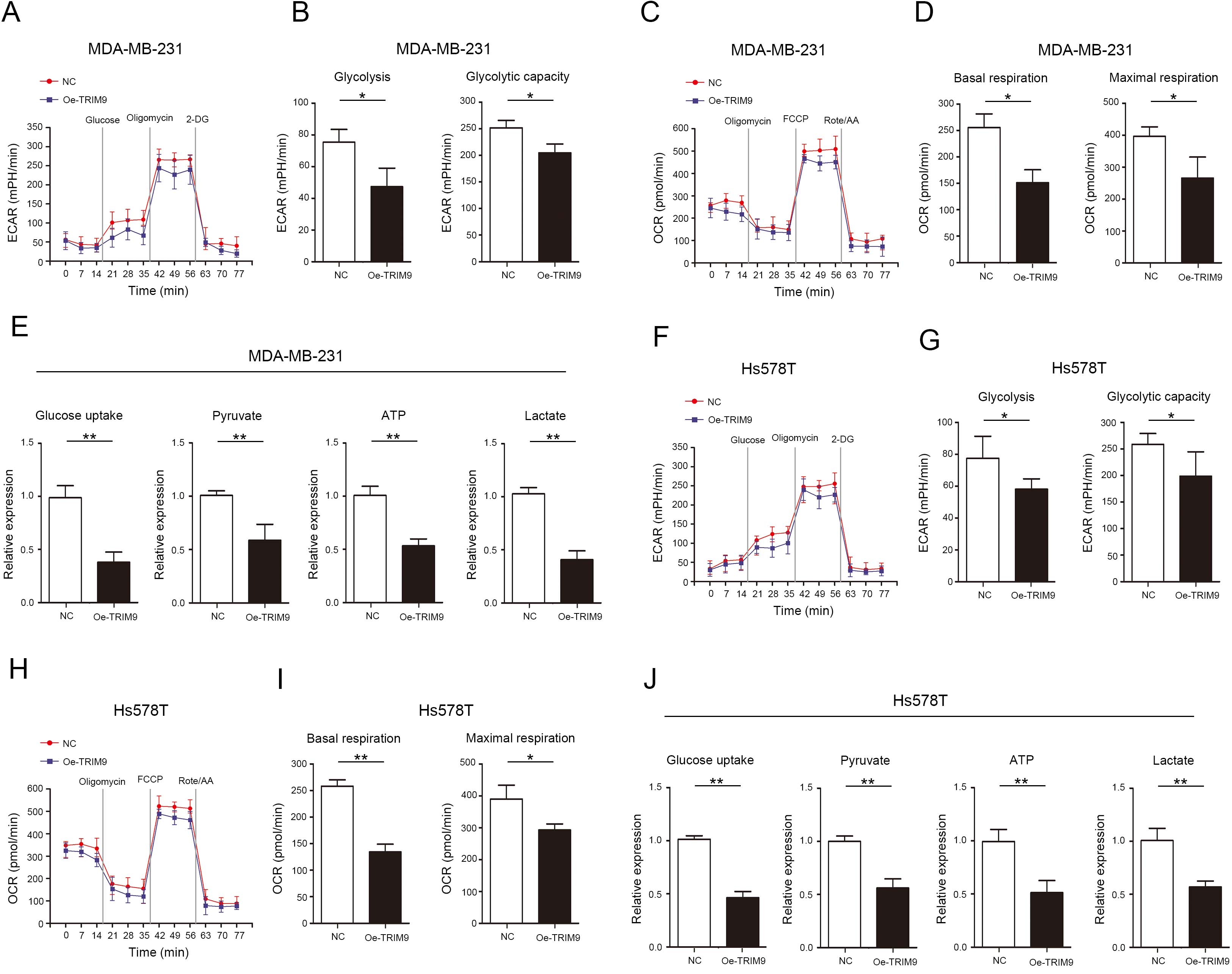

Supplement: Supplementary file 6 — Figure S6. [file JCMM-28-e18112-s006.jpg]
