# Supplementary material for: METTL14/miR‐29c‐3p axis drives aerobic glycolysis to promote triple‐negative breast cancer progression though TRIM9‐mediated PKM2 ubiquitination
Source: J Cell Mol Med. 2024 Jan 23;28(3):e18112. doi: 10.1111/jcmm.18112 (PMC10844685; doi:10.1111/jcmm.18112)
Supplement: Supplementary file 7 — Table S1. [file JCMM-28-e18112-s002.docx]

METTL14:

Forward Primer 5' – AGT GCCGAC AGC ATT GGT G- 3'

Reverse Primer 5' –GGA GCA GAG GTA TCA TAG GAA GC- 3'

TRIM9

Forward Primer 5' –GTG TGC GGC TCC TTC TAT CG- 3'

Reverse Primer 5' –GCT GTA TAG GCT CAT CTT GTC CA- 3'

GAPDH

Forward Primer 5' –GGA GCG AGA TCC CTC CAA AAT- 3'

Reverse Primer 5' –GGC TGT TGT CAT ACT TCT CAT GG- 3'

miR-29c-3p

Forward Primer 5' –ACA CTC CAG CTG GTA GCA CCA TTG AAA T- 3'

Reverse Primer 5' –TGG TGT CGT GGA GTC G- 3'

U6

Forward Primer 5' –CTC GCA TTG GCA GGA CTT ATA CT- 3'

Reverse Primer 5' –AAT CGT CAC GAA TCT GTG AGT C- 3'
